# Non-dispersive second-order charge recombination at mesoporous TiO_2_/adsorbed molecules/electrolyte interfaces

**DOI:** 10.1039/d6sc05541a

**Published:** 2026-10-07

**Authors:** Mitsuru Narita, Shogo Mori

**Affiliations:** a Division of Chemistry and Materials, Faculty of Textile Science and Technology, Shinshu University Ueda Nagano 386-8567 Japan 24hs305a@shinshu-u.ac.jp

## Abstract

The large surface areas of mesoporous metal oxide electrodes have been widely exploited for various applications including photocatalysis, electrosynthesis cells, dye-sensitized solar cells, and perovskite solar cells. Understanding interfacial electron transfer kinetics is essential for these applications. Charge recombination between electrons in the electrode and surface-adsorbed molecules has frequently been reported to exhibit highly dispersive kinetics. For more than two decades, these results have been interpreted primarily with electron trapping and de-trapping within TiO_2_. Here, we show that charge recombination at the molecular-anchored mesoporous TiO_2_/electrolyte interface follows a non-dispersive second-order rate law and exhibit little temperature dependence under appropriate conditions. Furthermore, we show that the same TiO_2_ displays dispersive recombination depending on the structure of adsorbed molecules and electrolyte cations, demonstrating that the dispersive recombination is not an intrinsic property of mesoporous TiO_2_ and cannot be attributed solely to intra-band charge traps. Instead, the results suggest that electronic coupling disorder of the molecules on the TiO_2_ surface is a major origin of the dispersion.

## Abstract 2

Transient absorption decays at molecule-anchored mesoporous TiO_2_ follow non-dispersive second-order recombination on insulating glass with electrolytes containing metal cations, but exhibit dispersive on FTO.

## Graphical abstract

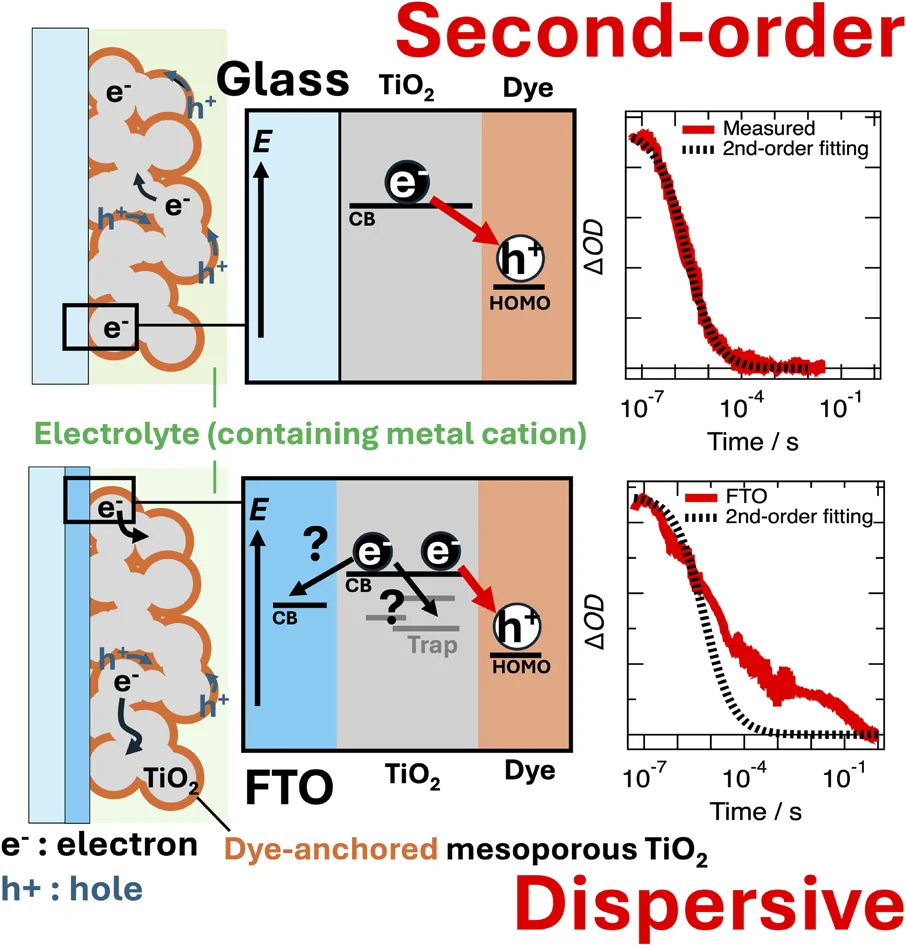

## Introduction

For electrodes in electrolyte solution, a large surface area of the electrodes per projected area is beneficial for charge separation,^1^ chemical reactions,^2^ and energy storage^3^ at the electrode/electrolyte interface. For photo-electrosynthesis cells and dye-sensitized solar cells (DSSCs), mesoporous electrodes can be fabricated easily by sintering metal oxide nanoparticles. Electron transport in the mesoporous electrode immersed in electrolyte solutions has been described as diffusion. This is because the particles are too small to form a depletion layer, and ions in the electrolyte screen the electric field in the electrode.^4–6^ Diffusion coefficients obtained by measuring currents induced by light intensity or electrical perturbation exhibited significant electron density dependence in the electrode.^7^ Among several proposed models, the random walk model with intra-band charge traps, known as the multiple trapping (MT) model, has been accepted widely.^5,8,9^ According to the model, apparent diffusion coefficients depend on the time to de-trap, which depends on the energy difference between the traps and the conduction band and the temperature of the electrodes. On the other hand, experimentally observed temperature dependence is not consistent with the MT model.^10,11^

For charge transfer from mesoporous TiO_2_ to molecules on the surface, the transfer process has been mostly monitored by a transient absorption (TA) method using a short-pulsed laser.^12^ The pulsed laser excites electrons in the molecules, and the excited electrons are injected into the conduction band of TiO_2_. Then, sometime later, injected electrons in the TiO_2_ transfer back to the holes in the molecules. Since the numbers of photogenerated electrons and holes are equal, second-order recombination is expected, as expressed below,1
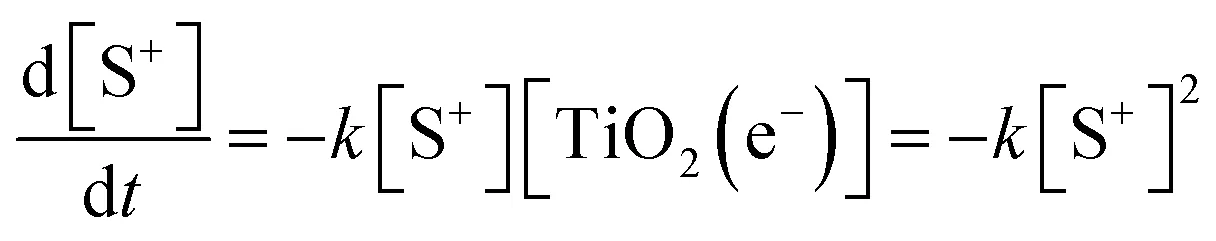
where *k* is the rate constant, [S^+^] is the concentration of oxidized molecules, and [TiO_2_(e^−^)] is the concentration of injected electrons in the TiO_2_. The reaction order was studied using an Ostwald isolation-type approach, which suggested a first-order dependence on both electron and hole densities.^13^ The solution of eqn (1) is2
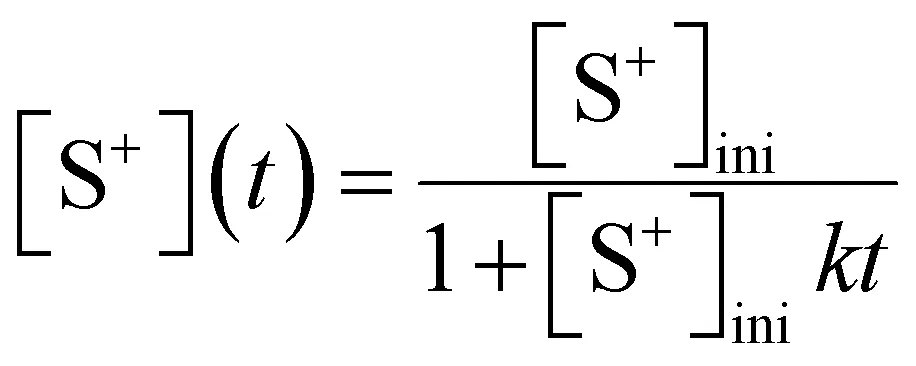
where [S^+^]_ini_ is the initial concentration of oxidized molecules. However, measured TA decays were frequently dispersive and fitted with a Kohlrausch–Williams–Watts (KWW) function,^14–16^

3 *A*(*t*) = *A*_0_e^−(*kt*)^*β*^^

where *A*_0_ is the initial amplitude of TA, *k* is the rate constant and *β* is related to the distribution of rate constants. The origin of the dispersive behavior remains unclear.^17^

The observed dispersive recombination kinetics has been modeled also with trap-limited diffusion, and the model has been widely accepted for more than 25 years.^18–20^ In the model, time to meet holes depends on the time electrons diffuse in the TiO_2_ electrode, and thus the recombination lifetime scales inversely with the apparent diffusion coefficients, which is limited by the event of trapping and de-trapping. Clifford *et al.* reported that TA decay for a porphyrin adsorbed TiO_2_ electrode became single exponential decay type recombination when the electronic coupling between the TiO_2_ and the highest occupied molecular orbital (HOMO) of the molecules was so weak that the limiting factor was the interfacial electron transfer.^21^ The half-time of the TA was about 100 ms. On the other hand, we have reported a slow and dispersive TA decay whose half-time was also about 100 ms.^22^ Thus, slow kinetics is not necessarily related to non-dispersive kinetics. Interfacial electron transfer kinetics depend also on how fast the holes in the molecules hop to neighboring molecules in the adsorbed molecule layer, and the dispersive kinetics would also occur due to energetic inhomogeneity of adsorbed molecules,^23^ making it difficult to identify the origin of dispersive behavior.

To study the recombination with the holes in the surface-anchored molecules, the mesoporous electrodes are usually fabricated on fluorine-doped tin oxide (FTO) transparent conductive glass. This is probably because mesoporous TiO_2_ was initially developed for DSSCs, which need FTO substrates. FTO is also useful to study the interfacial transfer kinetics because the desired potential can be applied to the mesoporous electrode to control the electron density in the electrode.^24^ On the other hand, using FTO influences the TA decay, requiring additional attention to analyze the TA.^25^ Katoh *et al.* measured TA for dyed TiO_2_ electrodes prepared on insulating glass substrates. They found that the TA decay half-time increased as the electron density decreased, even below one electron per TiO_2_ particle, which is not consistent with the trap-limited recombination model.^26^ Katoh and Furube also reported that the TA decay time constant was independent of temperature over a low temperature range and concluded that charge traps were not involved for the transfer kinetics.^27^ On the other hand, the TA decay they measured was not second-order type, and they speculated that it was caused by some heterogeneous surface conditions. Kelly *et al.* reported that the TA decays of Ru-dye-sensitized TiO_2_ films prepared on insulating glass substrates were consistent with second-order recombination kinetics over a range of excitation intensities.^28^ However, their transient absorption decays required the sum of two second-order equations for fitting, leaving open the possibility that the observed TA kinetics reflect a distribution of second-order rate constants.^17^

Although second-order recombination kinetics have been reported in some studies of mesoporous TiO_2_/molecule interfaces, no transient absorption decay has yet been shown to be fitted over the entire time range using a single second-order equation. Other studies have reported results inconsistent with the MT model. However, the reported kinetics were also dispersive.^27^ Because the origin of such dispersive kinetics has not been clearly identified, the MT model could not be excluded.^19,20^ In this study, we demonstrate that stretched exponential TA decay due to the charge recombination between injected electrons in the TiO_2_ electrode and holes in anchored molecules changes to a decay consistent with second-order recombination kinetics when an insulating glass substrate and metal cations in the electrolyte are simultaneously employed. In other words, mostly because previous studies did not employ insulating glass substrates and electrolyte solution at the same time, dispersive kinetics was observed. We also show the second-order like recombination for various molecules and cations, as well as several examples of dispersive kinetics, even with an insulating glass substrate and metal cations in the electrolyte. We then discuss the origins of the heterogeneous TA decay.

## Results

Scheme 1 shows the experimental setup and the structures of the molecules named MK-2, MK-1, MK-45, P251 and N719 examined here. In order to select appropriate probe wavelengths for the TA measurements, we measured the absorption spectra of oxidized dyes by photoinduced absorption spectroscopy (Fig. S1 in the SI). The similar spectral shapes in the absence and presence of LiClO_4_ suggest that electron injection occurs even when neat acetonitrile is employed and the observed TA decays correspond to the decay of oxidized dyes. We first compare the TA of MK-2 on about 0.5 µm TiO_2_ electrodes prepared on insulating glass substrates or FTO substrates immersed in acetonitrile with and without LiClO_4_ (Fig. 1). Regardless of the electrolyte, the TA decayed faster on the insulating slide glass substrates. On the insulating glass substrate, the TA decayed faster with LiClO_4_, while on FTO, the TA decay exhibited biphasic behavior, and the first phase decayed faster with LiClO_4_. For thicker films (∼3.2 µm) on FTO, the biphasic behavior was weakened, and the TA decayed more slowly when the molecules were excited from the FTO (substrate) side (Fig. S2 in the SI). For the thin film used for Fig. 1, the biphasic decays were observed for the samples on FTO regardless of the pulsed laser intensity under electrolyte side irradiation (Fig. S3 in the SI). Among the four conditions examined in Fig. 1, only the electrode prepared on the insulating glass substrates in the presence of LiClO_4_ displayed TA decays that were well fitted using eqn (2), suggesting second-order recombination kinetics. Fig. S4 in the SI shows TA for the same comparisons with a Ru complex dye called N719. Similar to the case of MK-2, the TA decay for N719 was faster on glass substrates and showed second-order like recombination in the cells using LiClO_4_ and slide glass substrates. In contrast to the case of MK-2, the effect of LiClO_4_ was small for N719/TiO_2_/glass cells. Fig. 1c shows pulse intensity dependent TA for MK-2/TiO_2_/glass with LiClO_4_ and Fig. 1d displays the half-time of the TA decays as a function of the density of injected electrons. All the TA decays were fitted with eqn (2) over a range of two orders of magnitude in electron density. The decrease in the half-time of TA over a wide range of electron densities is consistent with the report by Katoh *et al.*^27^Fig. 1d also includes the half-time of TA from MK-2/TiO_2_/FTO with LiClO_4_ samples, showing steeper dependence on the electron density and leveling off below around 1 × 10^17^ cm^−3^. The density of TiO_2_ particles was estimated to be 1.5 × 10^17^ cm^−3^ using the diameter of the particles. Such steep dependence and leveling off behavior at around one electron per TiO_2_ particle were reported by Haque *et al.* with a Ru complex dye named N3 and explained with trap-limited recombination.^24^ We replicated Haque's results for the similar Ru complex dye, N719, on TiO_2_ on an FTO substrate, while less steep dependence of the half-time on electron density over a wide range was observed on a glass substrate, similar to the case of MK-2, as shown in Fig S4(c). These results suggest that electron recombination at the TiO_2_/molecule interface is not limited by the electron transport in mesoporous TiO_2_ with trapping/de-trapping events but is influenced by FTO.

**Scheme 1:**
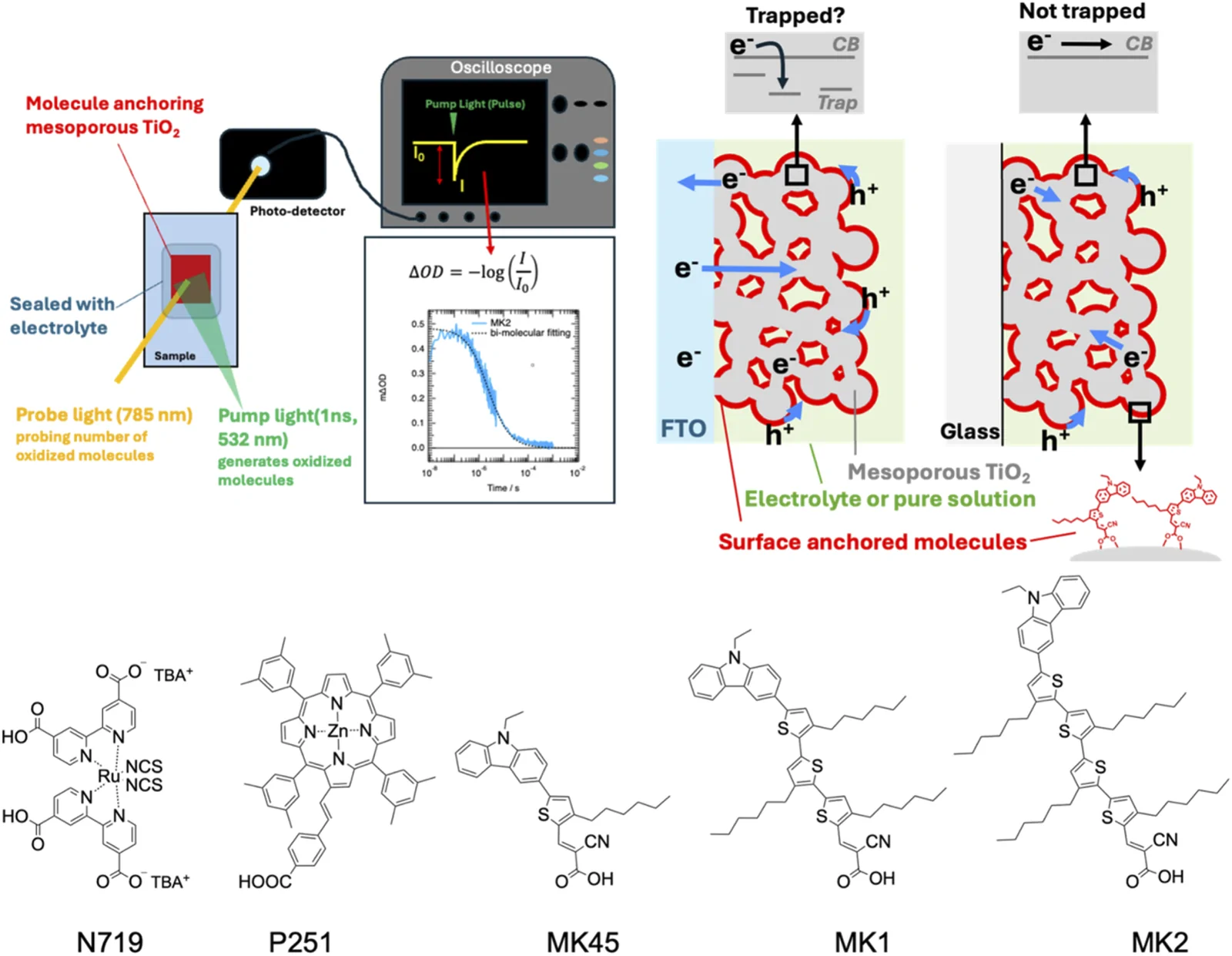
Measurement setup of transient absorption of laser pulse induced oxidized molecules, sample configurations and structures of molecules adsorbed on the mesoporous TiO_2_ electrode. A laser pulse generates the same number of injected electrons and holes in the TiO_2_ surface adsorbed molecules. While the second-order recombination kinetics is expected for this system, previous reports display highly dispersive kinetics, which has been attributed to trap limited recombination.

**Fig. 1:**
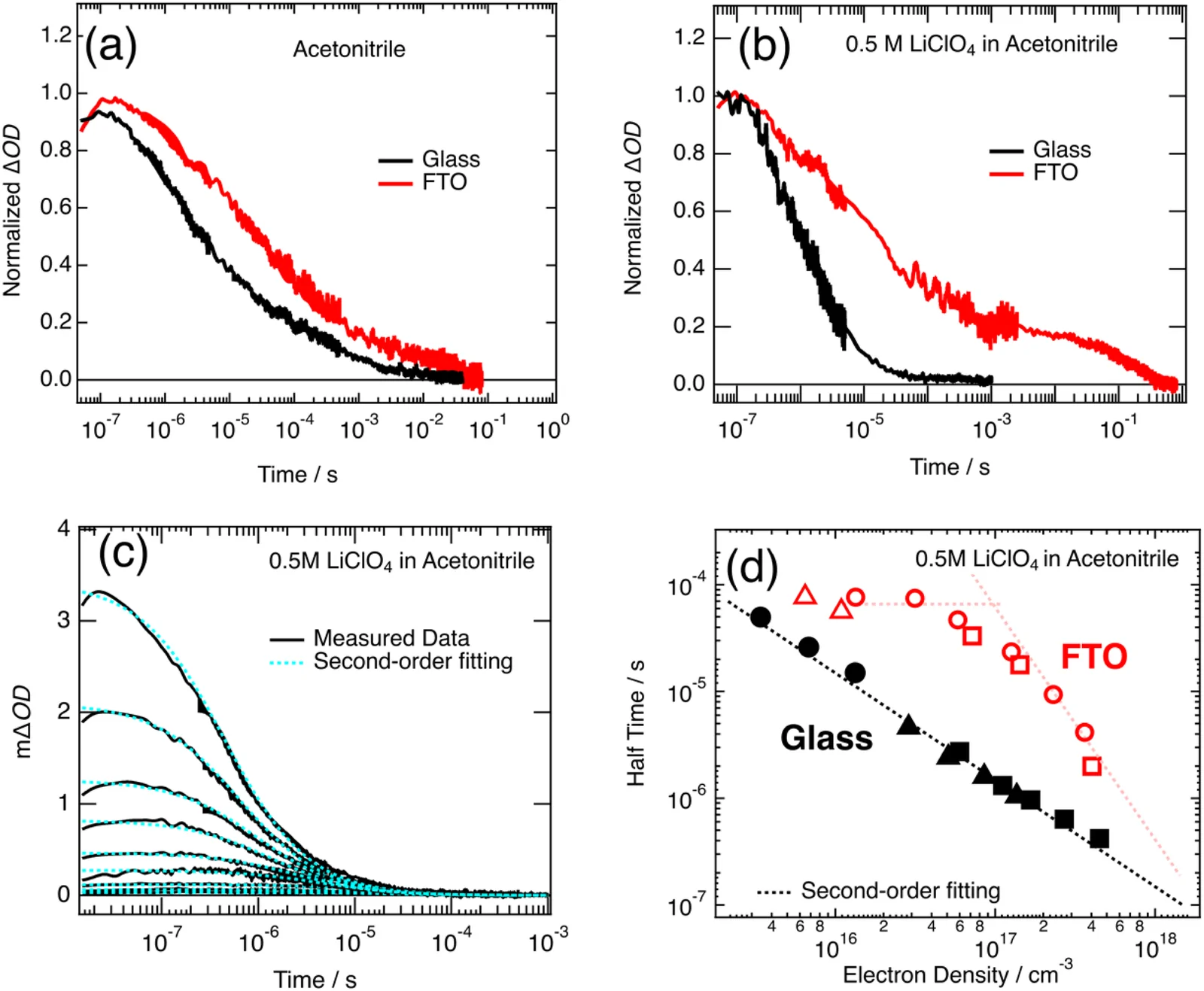
TA decays of MK-2 sensitized TiO_2_ electrodes immersed in only acetonitrile (a) and in acetonitrile containing 0.5 M LiClO_4_ (b), TA decays of the MK-2/TiO_2_ electrode on a glass substrate immersed in 0.5 M LiClO_4_ under various excitation pulse intensities (c), and the half-time of the TA decays as a function of injected electron density (d). For (a), (b) and (d), the TiO_2_ electrodes were deposited on insulating or FTO glass substrates. The thickness of TiO_2_ was about 0.5 µm. The pulsed laser was irradiated from the electrolyte side. Different symbols indicate different samples prepared under identical conditions.

We observed second-order recombination when LiClO_4_ was present. Such kinetics might appear because the adsorption of Li^+^ on the TiO_2_ surface masked the effect of traps. Then, we examined the effect of different electrolyte cations. Fig. 2 shows the TA decays of MK-2/TiO_2_ immersed in electrolyte with 0.5 M of various electrolytes. All cations except tetrabutylammonium (TBA^+^) exhibited second-order like recombination kinetics, while TBA^+^ showed a deviation from the second-order kinetics model at later time scales. Among the cations, Li^+^ and Na^+^ showed the fastest and slowest kinetics, respectively. Li^+^, Na^+^, and Mg^2+^ have been reported to be adsorbed on TiO_2_, but TBA^+^ has not.^28,29^ The measured absorption spectra of MK-2/TiO_2_ immersed in various electrolytes also indicate the adsorption of these cations, except for TBA^+^, whose spectrum was the same as that in acetonitrile without electrolytes (Fig. 2d). We also measured TA with 25 mM LiClO_4_ and found the second-order-like recombination (Fig. S5 in the SI). Therefore, it is less likely that the traps are masked similarly under all the various electrolyte conditions examined here, implying that there are few effective traps in mesoporous TiO_2_. The mesoporous electrode prepared from a paste of TiO_2_ particles purchased from Solaronix also showed nearly second-order recombination for MK-2 on insulating glass substrates with LiClO_4_ in acetonitrile (Fig. S6 in the SI), showing that the observation here is not a particular case for PST18-NR TiO_2_ particles. Finally, TA decays for a MK-2/TiO_2_/glass substrate immersed in electrolyte with and without 0.5 M LiClO_4_ were measured at three different temperatures (Fig. 3). The TA decay showed little temperature dependence in the absence of LiClO_4_, whereas a clear temperature dependence was observed in the presence of LiClO_4_. Temperature dependence can be caused by interfacial charge transfer described by Marcus theory and/or the frequency of trapping and de-trapping events. If recombination is limited by electron transport in TiO_2_, as described by the multiple-trapping model, temperature dependence will always be observed because waiting time *t* at a trap site is described by a Boltzmann distribution:^18^4
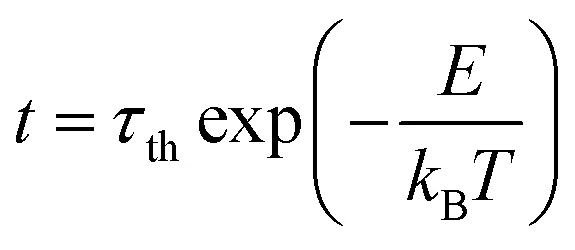
where *τ*_th_ is the minimum thermal activation time, *E* is the trap energy associated with each site, *k*_B_ is the Boltzmann constant, and *T* is the temperature. Interfacial electron transfer is often explained using the Marcus equation:5
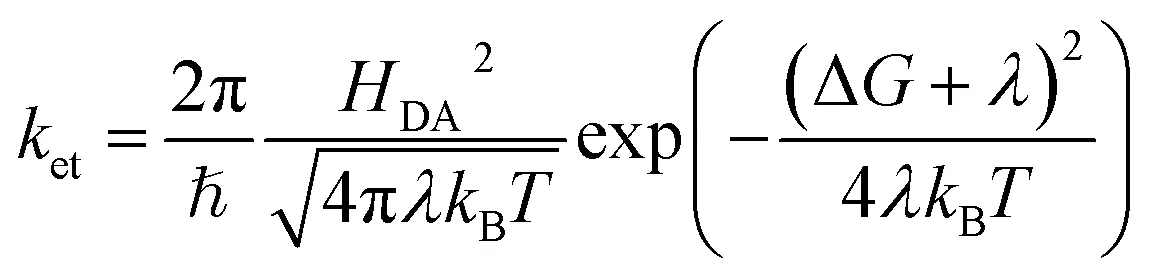
where *H*_DA_ is the electronic coupling between the donor and acceptor, Δ*G* is the Gibbs free-energy difference, and *λ* is the total reorganization energy; ℏ, *k*_B_, and *T* are the reduced Planck constant, Boltzmann constant, and temperature, respectively. If the recombination rate is limited solely by the interfacial electron transfer at the dye/TiO_2_ interface, as described by eqn (5), its temperature-dependence can be either pronounced or negligible (*e.g.*, only a 1.1-fold change over the temperature range studied here), depending on the Marcus region in which the recombination occurs.^30–32^ The little temperature dependence observed without LiClO_4_ suggests that few traps exist in TiO_2_, and the temperature dependence observed with LiClO_4_ can be attributed to a decrease in the free energy difference for the interfacial electron transfer. In other words, the results suggest that the recombination with and without LiClO_4_ occurs in the Marcus normal region and near the top of the Marcus parabola.

**Fig. 2:**
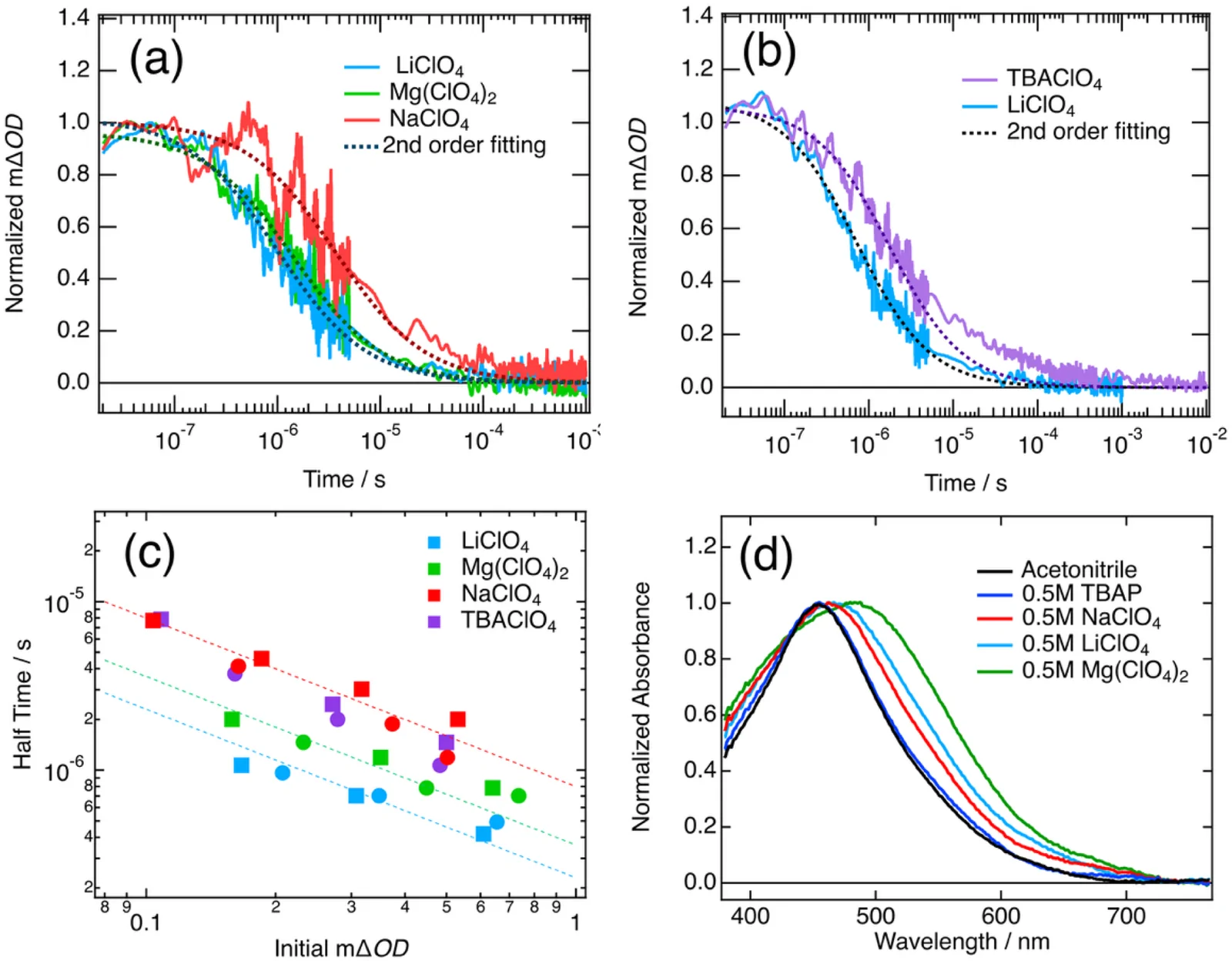
TA decays of MK-2 sensitized TiO_2_ electrodes on glass substrates immersed in acetonitrile containing 0.5 M LiClO_4_, Mg(ClO_4_)_2_ and NaClO_4_ (a); TA decays in acetonitrile containing 0.5 M TBAClO_4_ (b); and the half-time of the TA decays as a function of initial optical density difference (c). Different symbols with the same color indicate different samples prepared under identical conditions. Absorption spectra of MK-2 sensitized TiO_2_ electrodes immersed in various electrolytes (d). The values of ΔOD of each cell for (a) were 0.16 for Na^+^ and TBA^+^, 0.20 for Li^+^ and 0.24 for Mg^2+^.

**Fig. 3:**
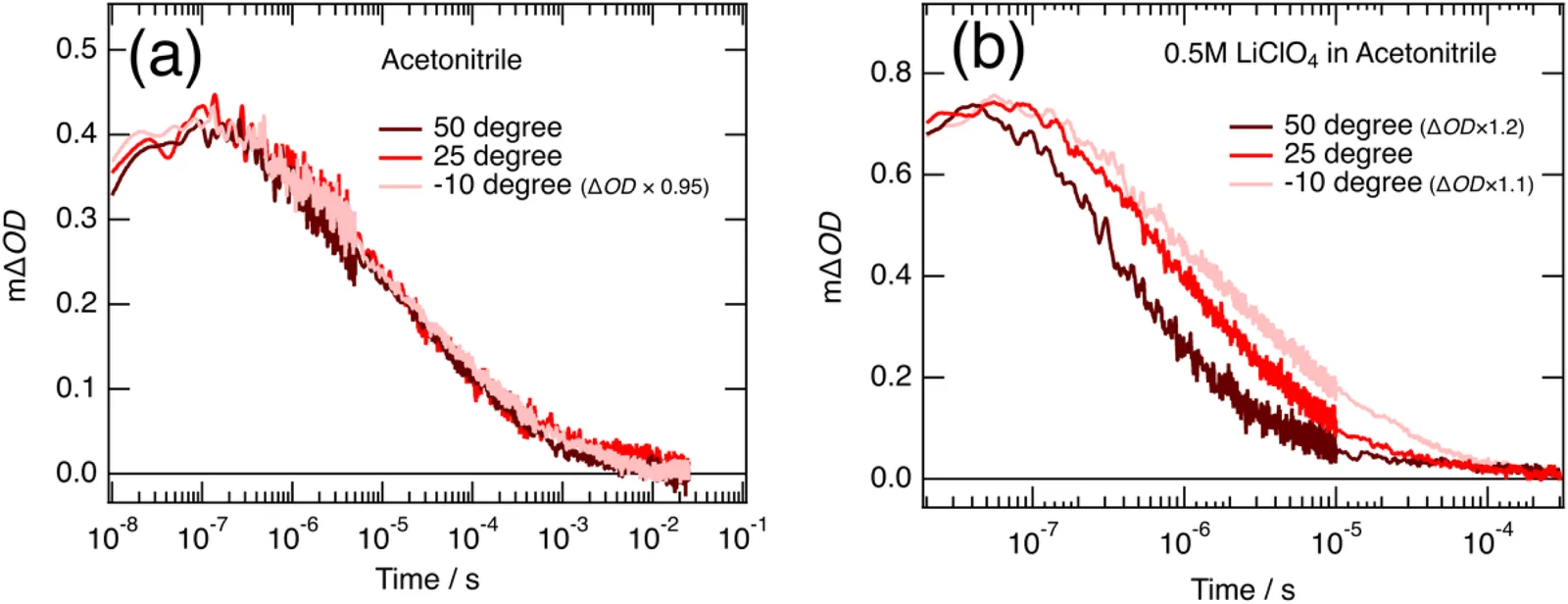
TA decays of MK-2 sensitized TiO_2_ electrodes on glass substrates immersed in acetonitrile (a) without and (b) with 0.5 M LiClO_4_, at three different temperatures.

Clifford *et al.* reported that a porphyrin dye showed single exponential TA decay, whose half-time is around 100 ms, and it was interpreted with the dye/TiO_2_ interfacial charge transfer limited case.^21^ For this case, the interfacial transfer was interpreted as a slower process than the trap limited diffusion. We observed second-order-like recombination at a much faster time scale, that is, faster than 10^−6^ s. The TA decay with Na^+^ in Fig. 2 was slower than that with Li^+^ but still nearly second-order recombination. Eng *et al.* showed an analytical function giving the trapping effect, which does not appear at a fast time scale because thermalization with traps is not reached, and thus the effect appears at a longer time scale.^33^ For the case of cations other than Mg^2+^, the tail of the TA signal deviates slightly from the fitted line described by eqn (2). While this deviation could in principle arise from trapping effects, we associate it primarily with less homogeneous surface conditions caused by the adsorbed cations, as discussed in the following section.

To examine the generality of the second-order recombination kinetics on the mesoporous TiO_2_ electrode, TA decays of three more dyes and TA of MK-2 under lower dye loading conditions were measured, as shown in Fig. 4. MK-1 showed nearly second-order recombination, but MK-45 and P251 displayed strongly deviated decays from eqn (2). The TA decays of MK-45 and P251 were almost the same between the conditions using the solutions with and without LiClO_4_ (Fig. S7 in the SI). These results show that whether the recombination follows second-order kinetics depends on the dye structure and adsorption density.

**Fig. 4:**
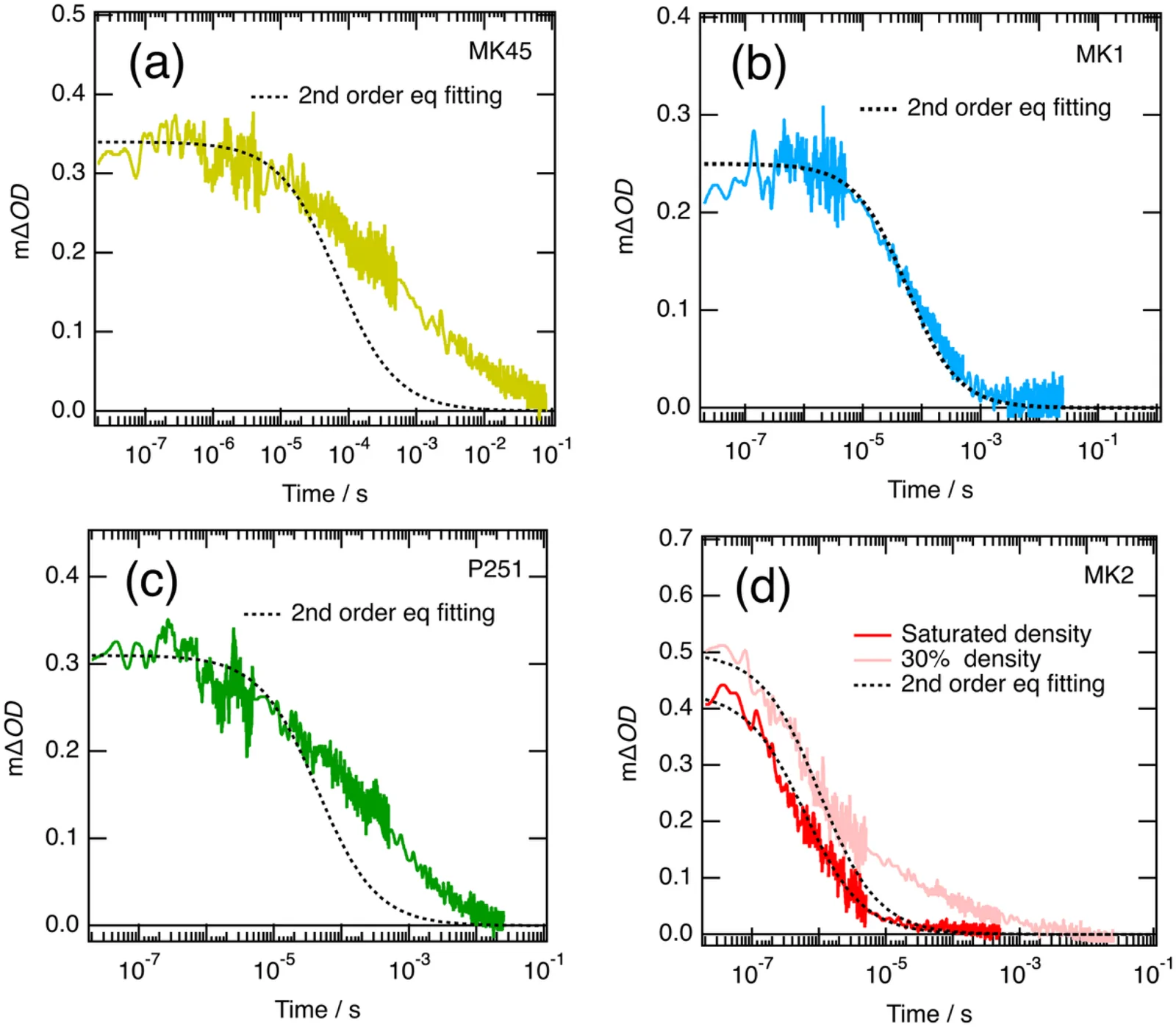
TA decays of MK-45 (a), MK-1 (b), P251 (c), and MK-2 (d) anchored TiO_2_ electrodes on glass substrates. The electrolyte was 0.5 M LiClO_4_ in acetonitrile. For MK-2, TA decays for the cells with different MK-2 coverage electrodes are shown. Different pulse intensities were used for the different coverage cells in (d). The saturated adsorption density was estimated for 430 µmol cm^−3^, and 30% of the saturated adsorption density was estimated from the absorption spectrum (Fig. S13 in the SI).

In DSSCs, the electrolyte contains redox species to regenerate (reduce) the photo-induced oxidized dyes. Injected electrons in TiO_2_ can transfer not only to oxidized dyes but also to the redox species in the electrolyte. Electron density dependent rates of electron transfer to redox species in solution have been observed and interpreted with trap limited electron transfer.^7,8^ To measure the transfer rate, or the value called the electron lifetime, the open circuit voltage response against perturbed light intensity is typically measured,^8^ and impedance spectroscopy^34^ and transient absorption can also be used.^35^ The metal-free oxidized organic molecules examined here have absorption in the infrared region, but N719 does not. In the case of N719, electrons in the conduction band of TiO_2_ can be monitored by measuring the absorption in the infrared region.^35^ Based on the results in Fig. 1 and S4, interfacial electron transfer is not limited by traps in the TiO_2_ electrode prepared on insulating glass. Thus, the electron transfer from TiO_2_ on the insulating glass substrates to redox species is also not expected to depend on the trap filling effect. Fig. 5 shows the TA of electrons in the N719 adsorbed TiO_2_ on FTO or insulating glass using the I^−^/I_3_^−^ redox couples and Li^+^ as the counter cation. Measurements were performed under open circuit conditions with and without bias light, which changed the steady state electron density in the TiO_2_ electrode. The cells prepared on insulating glass did not show the effect of the bias light on the TA decay, and the TA can be fitted with an exponential function. On the other hand, the cells using FTO showed the bias light intensity dependence. The observed lack of electron density dependence for TA on insulating glass under the examined light intensities is explained by the higher concentration of I_3_^−^ than that of electrons in the electrode without traps.

**Fig. 5:**
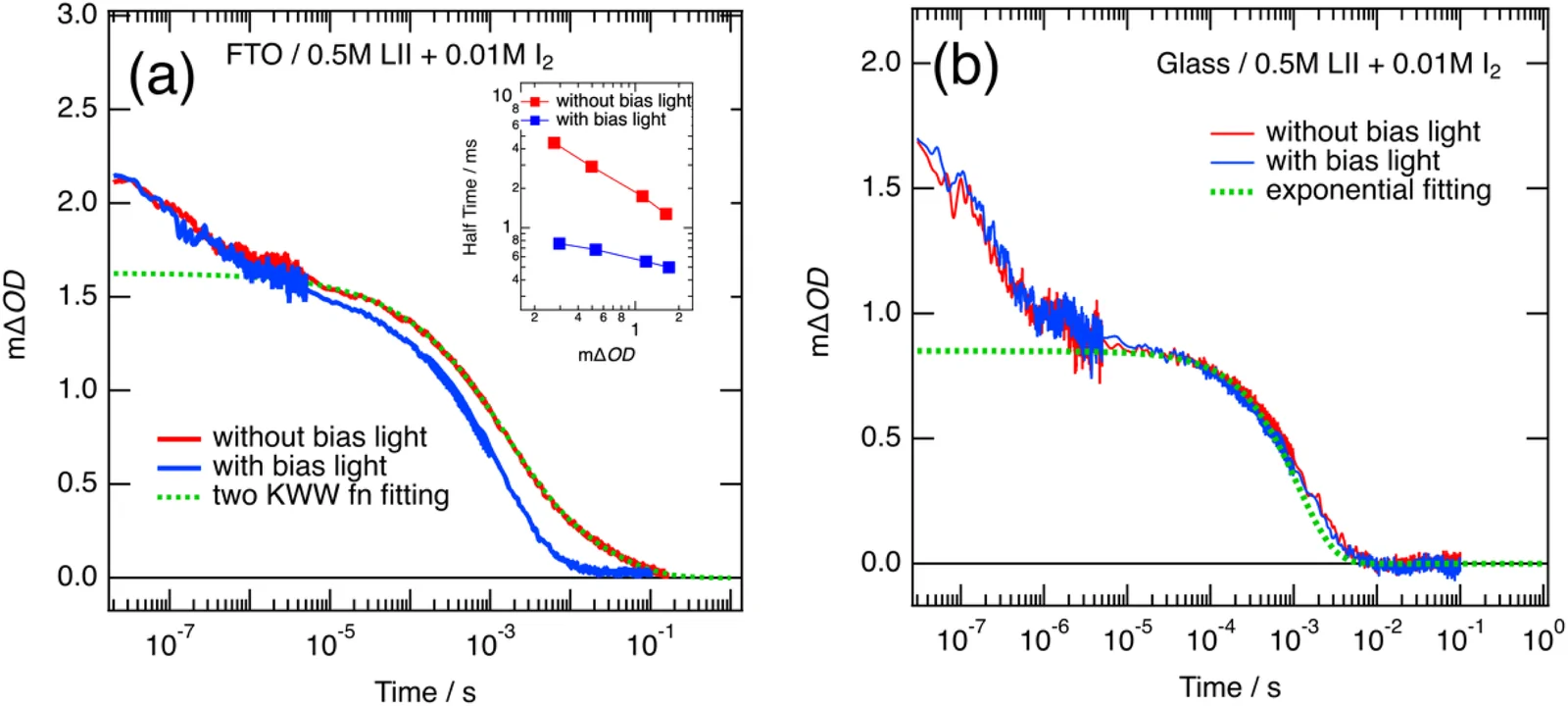
TA decays of a Ru complex, N719, sensitized TiO_2_ solar cells (a) and N719 sensitized TiO_2_ electrode sandwiched between two insulating glasses (b). The same electrolytes were used for (a) and (b). The probe wavelength was 980 nm, which detects only electrons in the TiO_2_ electrode. Measurements were performed with and without bias light. The inset of (a) shows the half-time of the TA as a function of initial ΔOD.

## Discussion

### Influence of the FTO substrate on the TA decay

Under light irradiation, electrons are injected into the TiO_2_ electrode and electrons diffuse in the electrode before they recombine with oxidized dyes. For the electrodes fabricated on FTO substrates, a possible explanation is that electrons move to the FTO and come back to the TiO_2_ electrode, causing extra time for recombination in comparison to the electrode on slide glass substrates. Fig. S2(a–c) shows a comparison of TA induced by pulse irradiation from the substrate or electrolyte side for MK-2 adsorbed TiO_2_ electrodes. The thickness of the TiO_2_ was about 3.2 µm. While the electrodes on FTO substrates showed the laser pulse irradiation direction dependent TA, the electrodes on slide glass did not show such dependence. On FTO substrates, when the pulse irradiated from the FTO side, the TA decay was slower than that induced by a pulse from the electrolyte side. This suggests that a fraction of electrons generated close to FTO enter FTO and require additional time to return to TiO_2_. In addition, the electron density in the TiO_2_ electrode is decreased, causing slower recombination. When the film thickness was increased to 6.5 µm, the TA induced from the electrolyte side on the FTO substrate was similar to the TA on the glass substrate (Fig. S2(d)). Since the penetration depth of the 532 nm laser pulse for the MK-2/TiO_2_ electrodes is a few microns, most of the injected electrons in the 6.5 µm thick electrodes did not diffuse to the FTO, resulting in kinetics similar to those observed on the glass substrate. In contrast, when the film thickness was only 0.5 µm, many of the generated electrons diffused to the FTO, so the TA decay was clearly affected by the substrate (Fig. S2(e)).

The effect of FTO shown in Fig. 1 and S4 was not only to slow the TA decay but also to cause dispersive TA decay. On FTO substrates, the more dispersive TA decay from FTO side irradiation suggests the presence of a barrier at the FTO/TiO_2_ interface. There is a conduction band energy difference between the FTO and TiO_2_.^36^ The dispersive TA decay could be caused by the fact that the Fermi level of FTO and TiO_2_ was not matched in the dark due to low minority carrier density of the TiO_2_, inducing a barrier for electrons back to TiO_2_ electrode, and the barrier height depends on the electron density in TiO_2_.

### Origin of dispersive TA

We found second-order recombination kinetics for dyed TiO_2_ electrodes on insulating glass substrates immersed in electrolyte solutions containing metal cations. This suggests that intra-band charge traps are not the limiting factor for the interfacial transfer rates in mesoporous TiO_2_ electrodes. On slide glass substrates and in the presence of Li^+^ in solution, MK-2, MK-1 and N719 adsorbed onTiO_2_ exhibited approximately second-order recombination kinetics, while MK-45 and P251 did not. This implies that the dispersive electron transfer is primarily caused by the heterogeneous electronic coupling between the molecules and TiO_2_, and the coupling becomes homogeneous when metal cations are adsorbed on TiO_2_ for particular dyes. Previously, we reported that the kinetics of hole hopping in the dye layer became more homogeneous when Li^+^ was added to solution and interpreted that the adsorbed Li^+^ reduced the energetic disorder of the dye.^22^ For MK-2 and MK-1, the second-order like recombination kinetics may be explained by the reduced energetic disorder with Li^+^.

When cations are adsorbed onto the TiO_2_ surface, anchored dyes experience the electric field generated by the cations, which can induce spectral shifts caused by modification of molecular orbitals^37^ and/or by the Stark effect.^38,39^ Spectra of the anchored dyes in acetonitrile with and without LiClO_4_ are shown in Fig. S8 in the SI. MK-2 and MK-1 showed red shift in the solution containing LiClO_4_, while MK-45, N719, and P251 displayed a little or no shifts. A correlation was seen between the magnitude of this spectral shift and the acceleration of the recombination rate with the addition of LiClO_4_ (Fig. S7 and S8). MK-2 and MK-1 exhibited faster decay, second-order kinetics, and spectral shifts in the presence of Li^+^, whereas N719 showed slight acceleration and MK-45 and P251 showed no acceleration with dispersive kinetics and little spectral shifts. For MK-2, Mg^2+^ induced a larger spectral shift than Li^+^, and the TA decay with Mg^2+^ was well fitted by the second-order model over the time range starting before 10^−7^ s (Fig. S9 in the SI). On the other hand, employing TBA^+^ did not induce the spectral shift, and the TA decay showed large deviation from the second-order model (Fig. 2b and d). The effects of Na^+^ on the shift and kinetics were between Li^+^ and TBA^+^. Adsorption of cations shifts the conduction band edge of TiO_2_, and the reported magnitude of the shift followed the order Mg^2+^ > Li^+^ > Na^+^ > TBA^+^,^40^ which is consistent with the degree of the spectral shift observed for the MK-2 dye. The deviation from second-order recombination kinetics increased progressively from Mg^2+^ to TBA^+^, implying that the weaker the electric field in the dye layer becomes, the larger the deviation from second-order kinetics becomes. This trend may be attributed to an increase in electronic coupling inhomogeneity as the electric field weakens. The weak effect of Li^+^ on MK-45 and P251 may indicate that the Li^+^ induced electric field was not strong enough to perturb these dyes. One hypothesis for MK-45 is that the dye would be small to experience the electric field generated by adsorbed cations on the TiO_2_ surface; that is, the height of the dye from the surface is too short, so that the electric fields from the adsorbed cations around the dye cancel each other (Fig. S10 in the SI). For P251, the difference from MK-1 and MK-2 is that the HOMO is distributed two-dimensionally on the ring and located far from the adsorption moiety (Fig. S10 in the SI), and it could alter the response to the electric field. On the other hand, the geometry of MK-1 and MK-2 would become stabilized/aligned under a strong electric field. N719 behaves differently from the other dyes, showing little Li^+^ induced spectral shift but exhibiting nearly second-order recombination kinetics in the presence of Li^+^ (Fig. S7 and S8 in the SI). Since N719 bound through two carboxyl groups, the adsorption geometry may be already stabilized without Li^+^ in comparison to others, giving less energetic disorder in the electronic coupling. However, these speculations require further substantiation.

When the surface coverage of adsorbed MK-2 was decreased, the TA decay became dispersive (Fig. 4d) even in the presence of Li^+^. Previously, we reported that the effect of solvent on outer-sphere reorganization energy appeared under low molecule loading conditions.^25^ Thus, if the density of surface molecules was reduced non-uniformly, then two phases in the TA decay would appear; that is, one is from non-solvent surrounded molecules and the other from solvent surrounded molecules. MK-2 is reported to aggregate on the surface,^36^ suggesting difficulty in being adsorbed on TiO_2_ uniformly. We also note that the second-order recombination kinetics were observed for electrodes prepared using freshly prepared dye solutions. Repeated use of the same dye solution may gradually reduce the dye loading, leading to a non-uniform environment for the adsorbed dyes and less pronounced behavior of second-order kinetics. Water contamination of the dye solution may also be another source of dispersive kinetics.

We also performed similar experiments for MK-2 adsorbed SnO_2_ and ZnO mesoporous electrodes on slide glass substrates (Fig. S11 in the SI). The TA decays of MK-2 adsorbed SnO_2_ electrodes with LiClO_4_ exhibited second-order like recombination kinetics while those of ZnO electrodes showed more dispersive kinetics. On the other hand, the dependence of half time of the TA decays on the initial optical intensity, which is proportional to the concentration of oxidized dyes, followed second-order like behavior for ZnO while that for SnO_2_ showed weak dependence. The effect of Li^+^ on the TA decay of MK-2 adsorbed SnO_2_ was negligible. This can be explained by the fact that SnO_2_ electrodes hardly adsorb electrolyte cations. ZnO electrodes are more complicated because the surface may be dissolved by adsorption of the dyes through their carboxylic acid groups. Based on these preliminary results, the effect of charge traps on the recombination kinetics of mesoporous SnO_2_ and ZnO electrodes could not be clearly identified. Moreover, clear second-order recombination kinetics was not observed, possibly because of the difficulty in controlling the surface electric field on these electrodes.

## Conclusions

We found that commonly observed dispersive interfacial electron transfer kinetics for molecules anchored to mesoporous TiO_2_ electrodes fabricated on transparent conducting oxide substrates became second-order like recombination kinetics for electrodes on insulating glass substrates immersed in electrolyte solutions containing metal cations. The observed second-order like recombination can be interpreted without assuming intra-band charge traps. On slide glass substrates, MK-1, MK-2 and N719 adsorbed on TiO_2_ showed second-order like recombination but MK-45 and P251 did not. This implies that the dispersive electron transfer occurs due to inhomogeneous electronic coupling between the dyes and TiO_2_, and the coupling becomes homogeneous under a strong electric field for the surface-anchored molecules that can respond to the electric field. Conditions of surrounding solvent molecules may differ among the surface adsorbed molecules, giving another reason for dispersive electron transfer kinetics. Therefore, greater attention should be paid to these interfacial conditions to control the electron-transfer rate.

## Methods

Mesoporous TiO_2_ electrodes were prepared from a paste (PST18-NR, JGC Catalysts and Chemicals Ltd) by the doctor-blade method on either an FTO glass substrate (SnO_2_:F, NSG TEC10, Nippon Sheet Glass Co., Ltd) or insulating slide glass. A field emission scanning electron microscopy (FE-SEM) image of the surface of mesoporous TiO_2_ film is shown in Fig. S12 in the SI. The paste was diluted with ethanol to control the thickness. For example, a 20% dilution was used to fabricate about 0.5 µm thick electrodes. The film thickness was determined using a surface profiler (Dektak6MDS, Veeco; ET4000A, Kosaka Laboratory Ltd.), and examples of the film profile are shown in the SI. The films were dried at 120 °C for 10 min and subsequently sintered at 450 °C for 30 min in air. The size of the electrode was 2 × 2 mm.

SnO_2_ and ZnO pastes were prepared from nanoparticle colloidal dispersions purchased from CIK NanoTek. For the SnO_2_ paste, polyethylene glycol (PEG) with molecular weights of 20 000 and 500 000 was added at 20 wt% and 15 wt%, respectively, to the dispersion. The resulting films were sintered at 450 °C for 30 min in air after they were dried at 120 °C for 10 min.

MK-2 and N719 were purchased from Sigma-Aldrich and Solaronix. MK-45, MK-1 and P251 were synthesized based on published papers.^41–43^ Dye solutions (0.3 mM) were prepared using toluene for MK-2 and MK-1, ethanol for MK-45, and a 1 : 1 ratio of acetonitrile/*t*-BuOH for N719. P251 was dissolved in tetrahydrofuran. The electrodes were immersed in the dye solutions for 18 hours. The average amount of adsorbed MK-2, prepared by immersion for 18 hours, was determined to be 430 µmol cm^−3^ by desorbing the dye into a TBAOH solution (Fig. S13 in the SI). With the surface area of the electrode,^44^ the density of adsorbed MK-2 on the TiO_2_ surface is estimated to be 1.8 nm^−2^. The cells were made by placing a slide glass or a Pt-sputtered FTO with a thermal adhesive film. Electrolyte compositions are detailed in the respective figure captions. To obtain the relationship between the optical density of oxidized molecules on TiO_2_ and injected electron density, cells were fabricated using 0.5 M LiI and 0.01 M I_2_ in acetonitrile.

Photoinduced absorption spectroscopy (PIA) was performed for dye-adsorbed TiO_2_ films deposited on glass substrates in the absence and presence of 0.5 M LiClO_4_ in acetonitrile. Details of the measurement setup have been published elsewhere.^45^ These cells were irradiated by an LED (*λ*_max_ = 460 nm) with a square-wave-modulated input voltage. Irradiation and non-irradiation times were set to 1.0 and 2.0 s, respectively. The ΔOD was measured at every 10 nm, and the spectral resolution of the probe wavelength was 2.5 nm (reciprocal linear dispersion = 5 nm mm^−1^; slit width = 0.5 mm).

For transient absorption measurements, a pulsed laser (FWHM < 1.2 ns, 532 nm, FDSS-532-150, CryLas GmbH) was used to excite molecules on the TiO_2_ electrodes. The laser pulse profile has a slight tail extending up to approximately 20 ns after the main peak (Fig. S14). This causes a small amount of additional electron injection, and when this injection overlaps with the recombination process, it can cause deviations for TA decay from eqn (2). This deviation becomes more noticeable at high light intensities, where recombination occurs on a very early timescale. When the light intensity is low, the TA decay more closely follows second-order-like behavior, as shown in Fig. S14. The laser pulse was expanded by a lens to ensure irradiation of the entire TiO_2_ electrode area. The pulsed laser was irradiated from the substrate side except for a few cases in Fig. S2. The intensity of the laser was controlled by using neutral density filters. Two polarizers were placed to irradiate the laser pulse in a magic angle (54.7°) configuration. The repetition rate of the pulsed laser was set to be 11 Hz or lower. To probe the time-course change of absorption of oxidized dyes, continuous wave diode lasers (CUBE, Coherent) were employed. The probe wavelength was 786 nm for MK-2, MK-1, and N719, and 660 nm for MK-45 and P251. These probe wavelengths were selected based on the ground state and excited state absorption spectra of these dyes (Fig. S1 and S8 in the SI). The intensity of the probe laser was monitored using a detector (Model 1623, New Focus), an amplifier (BX-31A, NF Electronic Instruments, or DHPVA-100, Femto Messtechnik GmbH), and an oscilloscope (HDO4034 Teledyne Lecroy). In front of the detector, a notch filter (532 nm) and a band pass filter (785 or 660 nm) were placed. The monitored probe signal was averaged over 512 or 1024 repetitions to enhance the signal-to-noise ratio. The difference in optical density (ΔOD) of the molecules anchored to TiO_2_ between the OD without pulse irradiation and that after irradiation was calculated as −log(*I*(*t*)/*I*_base_), where *I*(*t*) is the probe light intensity at time *t* and *I*_base_ is the intensity without the laser excitation. Measurements were performed under open circuit conditions for the cells. To select the wavelength of the probe light, photo-induced absorption spectra of molecules adsorbed on the TiO_2_ electrode were measured using a homemade setup. The wavelength was selected to detect mostly oxidized molecules or electrons in TiO_2_. Temperature-dependent transient absorption measurements were conducted using a cryostat (CoolSpek, UNISOKU) over a temperature range of −10 °C to 50 °C. This range was selected to avoid both evaporation and freezing of the acetonitrile solvent. Temperature control was achieved *via* liquid-nitrogen. To ensure accurate sample temperature measurement, a handheld thermometer (DP-350, RKC Instrument Inc.) equipped with an external sensor (ST-51S-100-C, RKC Instrument Inc.) was employed, with the sensor attached directly to the sample.

To obtain the correlation between ΔOD and injected electron density, dye-sensitized solar cells were made using FTO substrates and an I^−^/I_3_^−^ redox couple in the electrolyte solution. The electrolyte solution was 0.5 M LiI and 0.01 M I_2_ in AN. Since the same cation and concentration as those used for the measurements in Fig. 1d and S4(c) were used, the spectrum of the oxidized dyes and the conduction band edge of TiO_2_ were expected to be similar. Note that this specific measurement was performed under short-circuit conditions, unlike the other TA measurements. The cells were connected to the oscilloscope at 50 ohm to measure current transient induced by the pulsed laser. Simultaneously, TA was measured. The electron density was obtained by integrating the current transient and divided by the electrode volume. Then the charge density was plotted against the maximum ΔOD just after pulse irradiation. To measure the charge transfer to redox species *i.e.* I_3_^−^, a 980 nm probe laser (OBIS, Coherent) was employed. Measurement was performed with and without the irradiation of a halogen light (JCR15V-150WBN) as a bias light.

For the HOMO shown in Fig. S10, theoretical calculations were performed on Gaussian 16 at the B3LYP/6-31g(d) level of theory.^46,47^ The structure optimization of the dyes was carried out without any symmetry constraint in the gas phase. The HOMO was visualized using GaussView 5.

## Author contributions

MN conceived the project, designed and performed all experiments, validated and visualized the data, and wrote the original draft of the manuscript. SM contributed to the formal analysis, supervised the project, and reviewed and edited the manuscript. MN and SM acquired funding for this work.

## Conflicts of interest

The authors declare no competing interests.

## Supplementary Material

SC-OLF-D6SC05541A-s001

## Data Availability

Datasets are available from the corresponding author upon reasonable request. The data supporting this article have been included as part of the supplementary information (SI). Supplementary information: photoinduced absorption spectra, additional TA decays and half-time analyses (effects of irradiation direction, substrate, film thickness, excitation intensity, LiClO_4_ concentration, TiO_2_ source, electrolyte cations and dyes), absorption spectra in different electrolytes, DFT-calculated HOMOs, TA data for SnO_2_ and ZnO electrodes, film characterization (surface profiles and FE-SEM image), dye adsorption densities, and the time response of the TA setup. See DOI: https://doi.org/10.1039/d6sc05541a.
