# Supplementary material for: Non-dispersive second-order charge recombination at mesoporous TiO2/adsorbed molecules/electrolyte interfaces
Source: Chem Sci. 2026 Oct 7. Online ahead of print. doi: 10.1039/d6sc05541a (PMC13682529; doi:10.1039/d6sc05541a)
Supplement: Supplementary file 1 [file SC-OLF-D6SC05541A-s001.pdf]

This journal is © The Royal Society of Chemistry

Supporting Information for

Non-dispersive second-order charge recombination at mesoporous TiO_2/adsorbed molecules/electrolyte interfaces

Mitsuru Narita* and Shogo Mori
Division of Chemistry and Materials, Faculty of Textile Science and Technology, Shinshu University, Ueda, Nagano 386-8567, Japan
*Corresponding author: Mitsuru Narita, Email: 24hs305a@shinshu-u.ac.jp

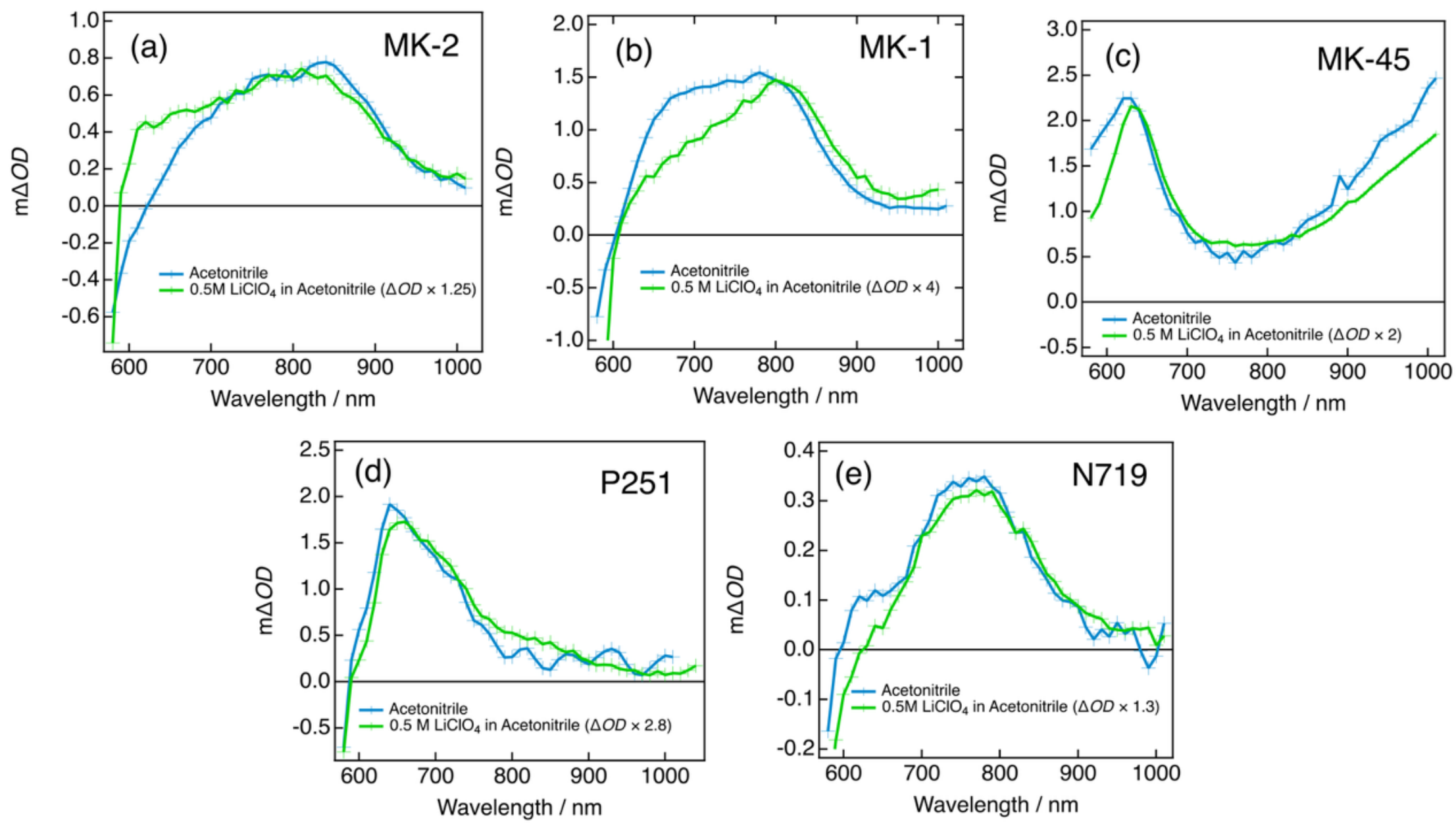

Figure S1. Photoinduced absorption spectra of the dye-anchored TiO_2 films examined in this paper. Green and blue lines represent spectra in the presence and absence of 0.5 M $LiClO_4$, respectively.

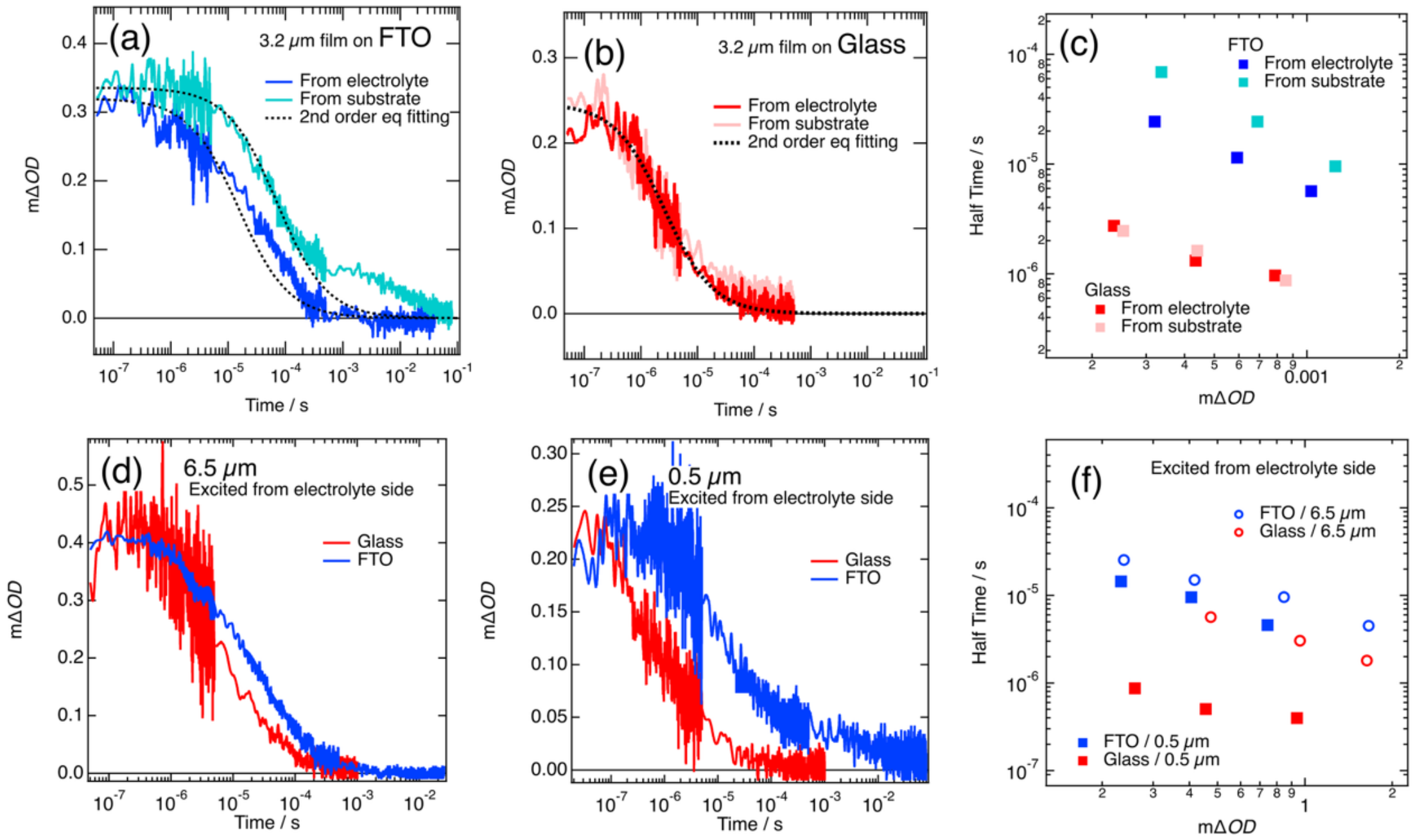

Figure S2. Effect of the pulsed laser irradiation direction on TA decay for the MK-2 sensitized electrodes fabricated on (a) FTO substrates, (b) glass substrates, and (c) the half-time obtained from the TA decays on different substrates and different irradiation directions as a function of the initial optical density difference. Thickness of the TiO_2 electrodes was about 3.2 µm. Effect of the substrate on (d) thick (6.5 µm), (e) thin (0.5 µm) MK-2 sensitized TiO_2 films, and (f) the half-time obtained from the TA decays from (d) and (e). Electrolyte was 0.5 M $LiClO_4$ in acetonitrile.

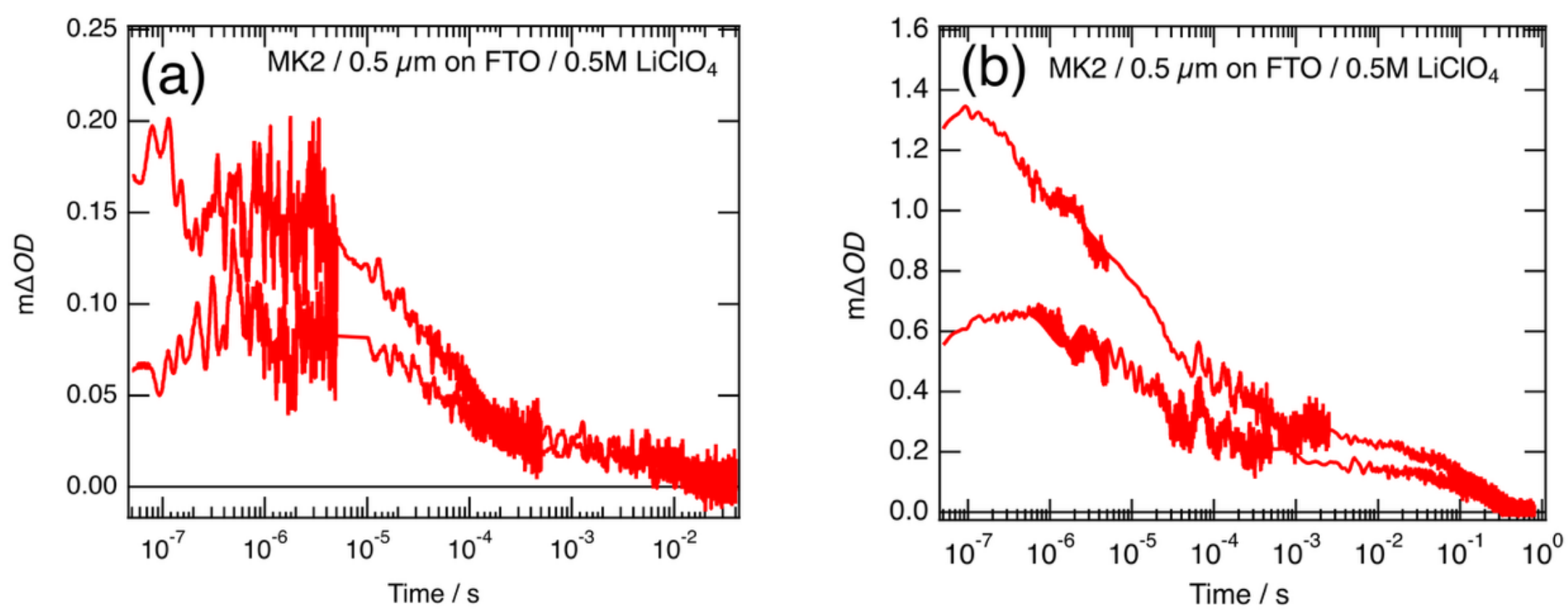

Figure S3. TA decays of MK-2 sensitized TiO_2 electrodes fabricated on FTO, immersed in 0.5 M $LiClO_4$ in acetonitrile. The excitation laser intensities were low (a) and relatively high (b). All samples were excited from the electrolyte side. Each graph shows two decays under two different pulse intensities.

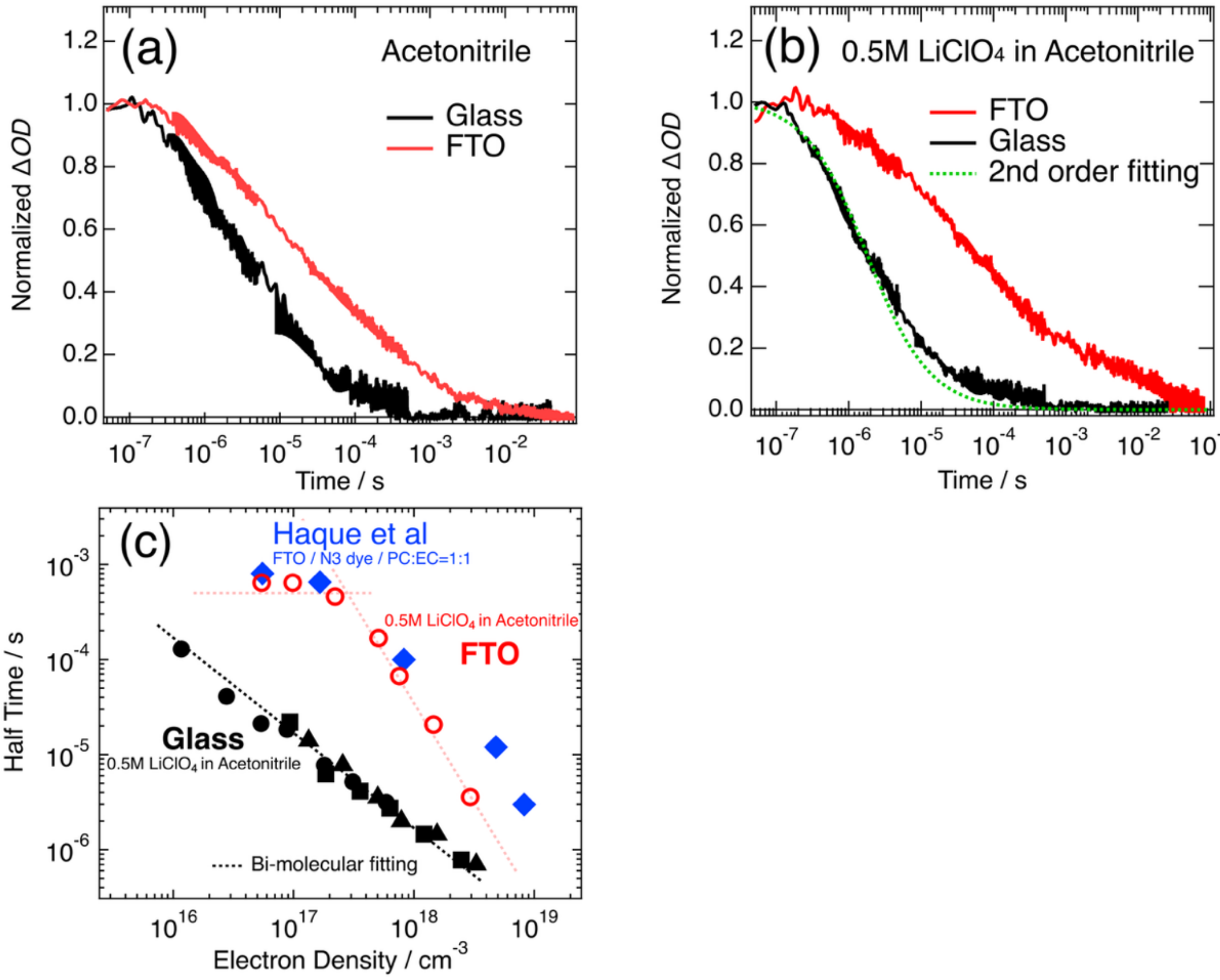

Figure S4. TA decay of N719 sensitized TiO_2 electrode immersed in acetonitrile (a) and in acetonitrile containing 0.5 M $LiClO_4$ (b), and half-time as a function of injected electron density (c). The different symbols with the same color indicate different samples prepared under identical conditions. In (c), data points extracted from a paper by Haque et al. (ref 24) were also plotted.

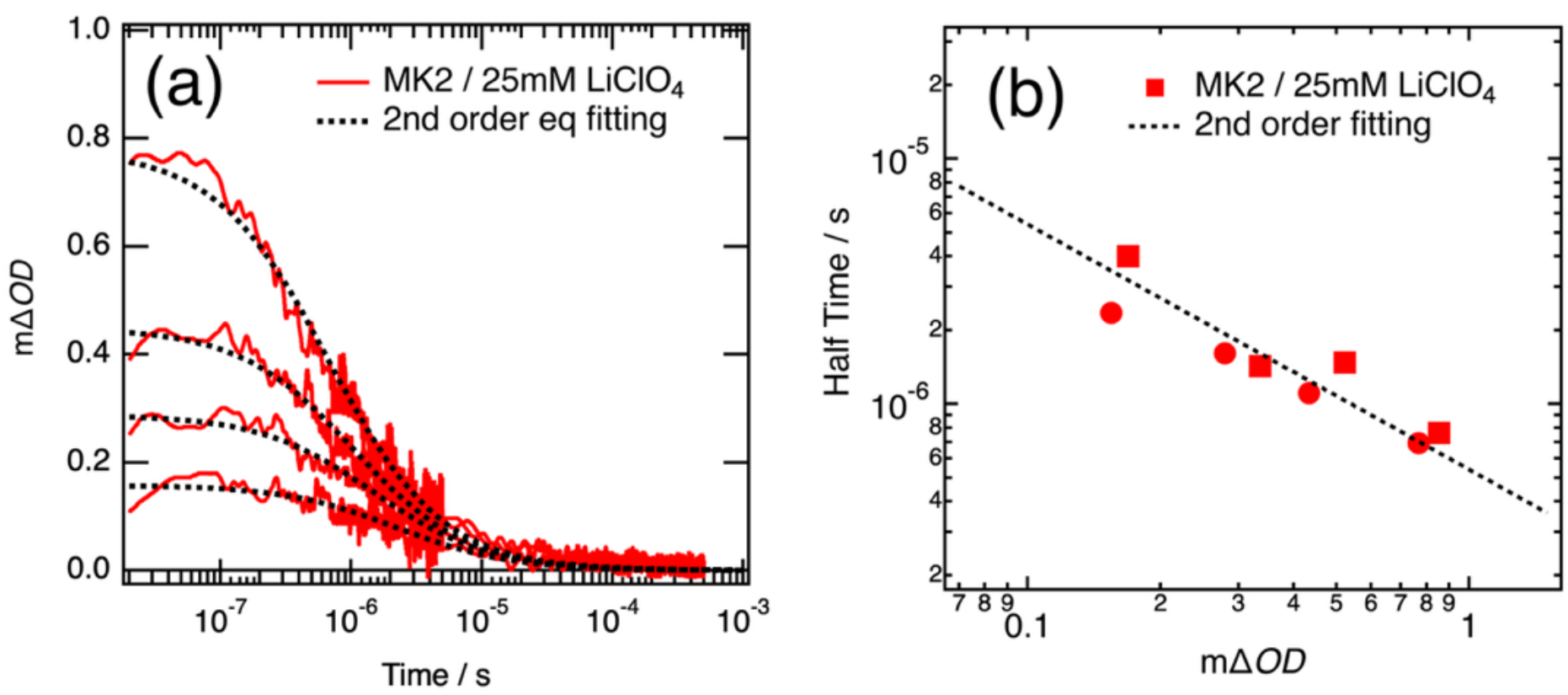

Figure S5. TA decays of MK-2 sensitized TiO_2 electrodes immersed in 25 mM $LiClO_4$ in acetonitrile (a) and the half-time of the TA decays as a function of initial ΔOD (b). The different symbols indicate different samples prepared under identical conditions.

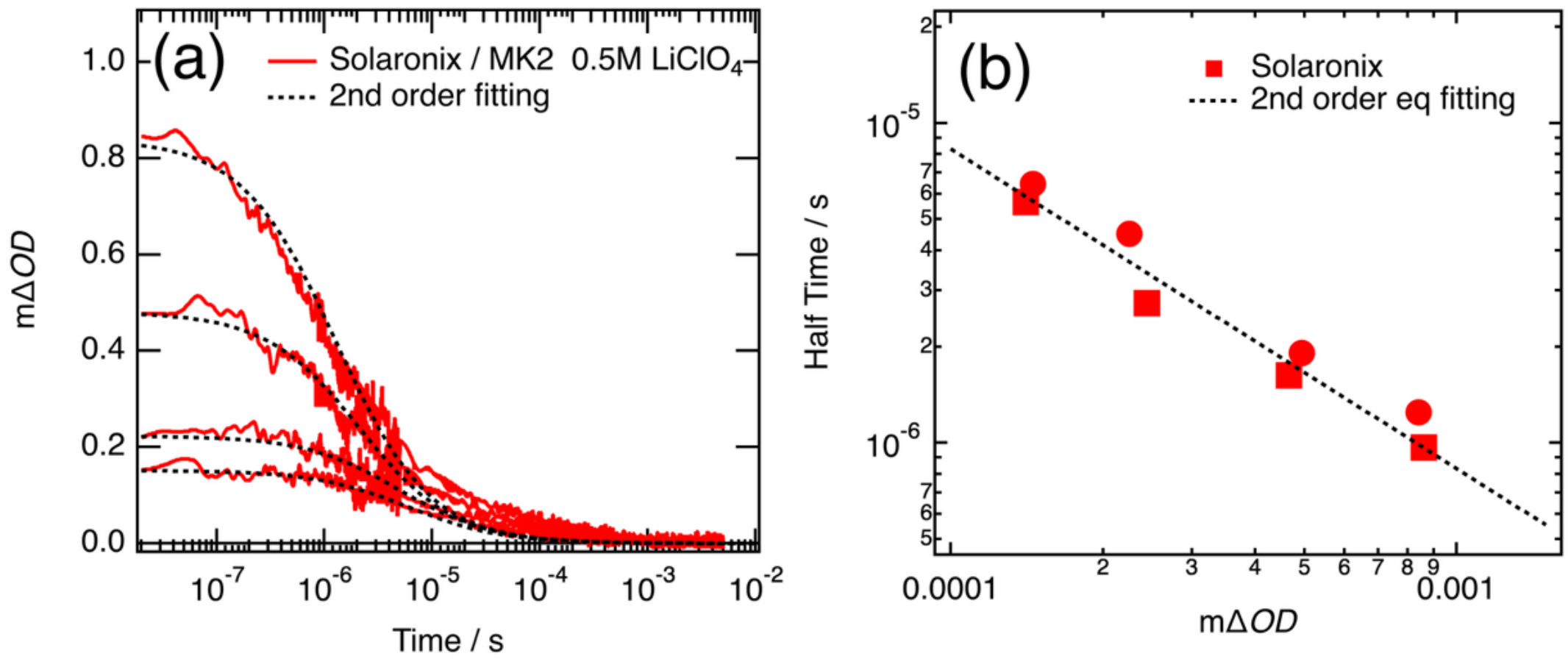

Figure S6. TA decays of MK-2 sensitized TiO_2 (Solaronix) electrodes immersed in 0.5 M $LiClO_4$ in acetonitrile (a) and the half-time of the TA decays as a function of initial Δ*OD* (b). The different symbols indicate different samples prepared under identical conditions. The electrodes were prepared on insulating glass substrates by annealing at 450 °C for 30 min in air. The thickness was about 1.9 μm.

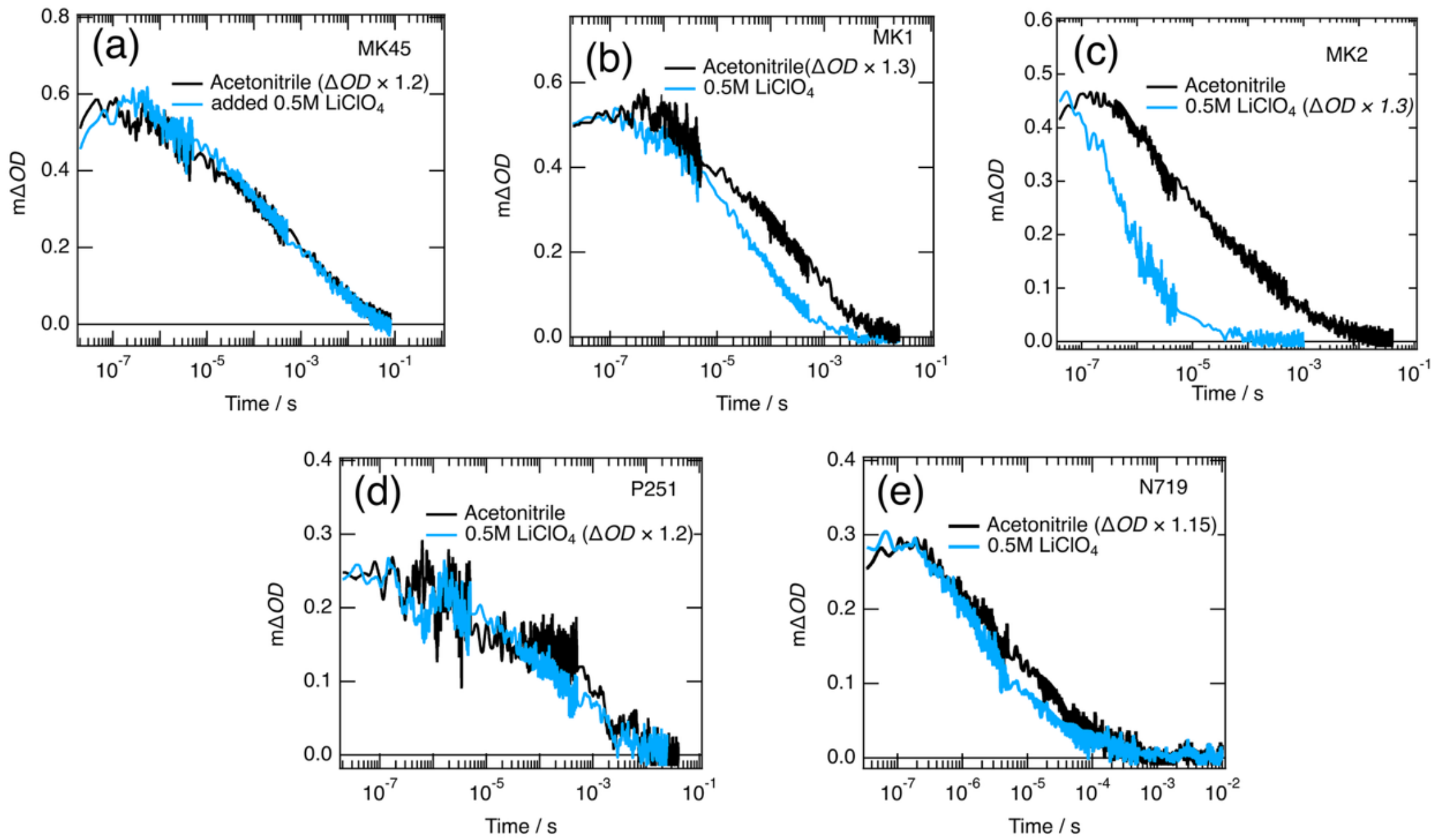

Figure S7. TA decays of (a) MK-45, (b) MK-1, (c) MK-2, (d) P251, and (e) N719 sensitized TiO_2 electrodes on glass substrates immersed in acetonitrile or 0.5 M $LiClO_4$ in acetonitrile.

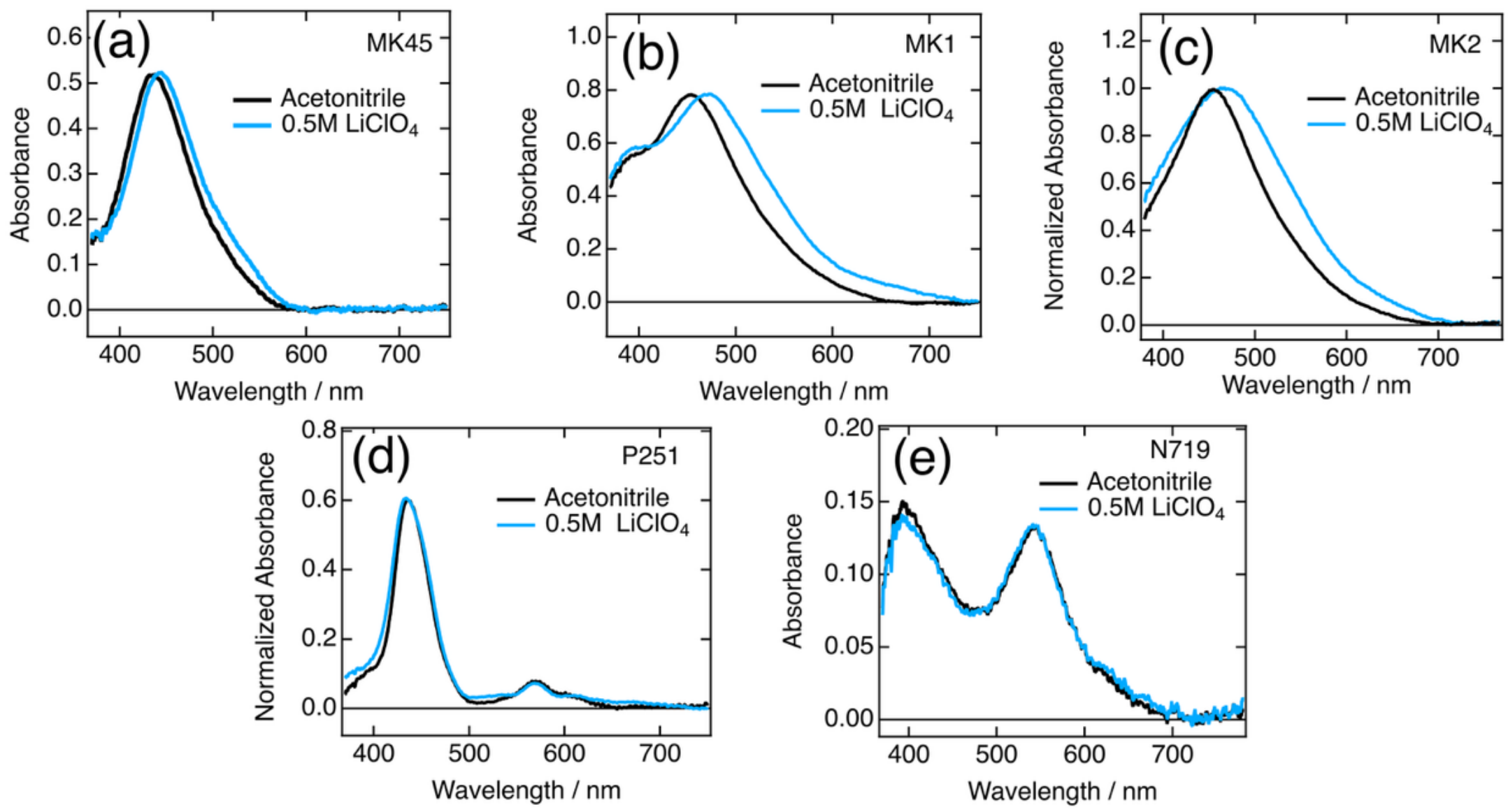

Figure S8. Absorption spectra of (a) MK-45, (b) MK-1, (c) MK-2, (d) P251, and (e) N719 sensitized TiO_2 electrodes immersed in acetonitrile or 0.5 M $LiClO_4$ in acetonitrile.

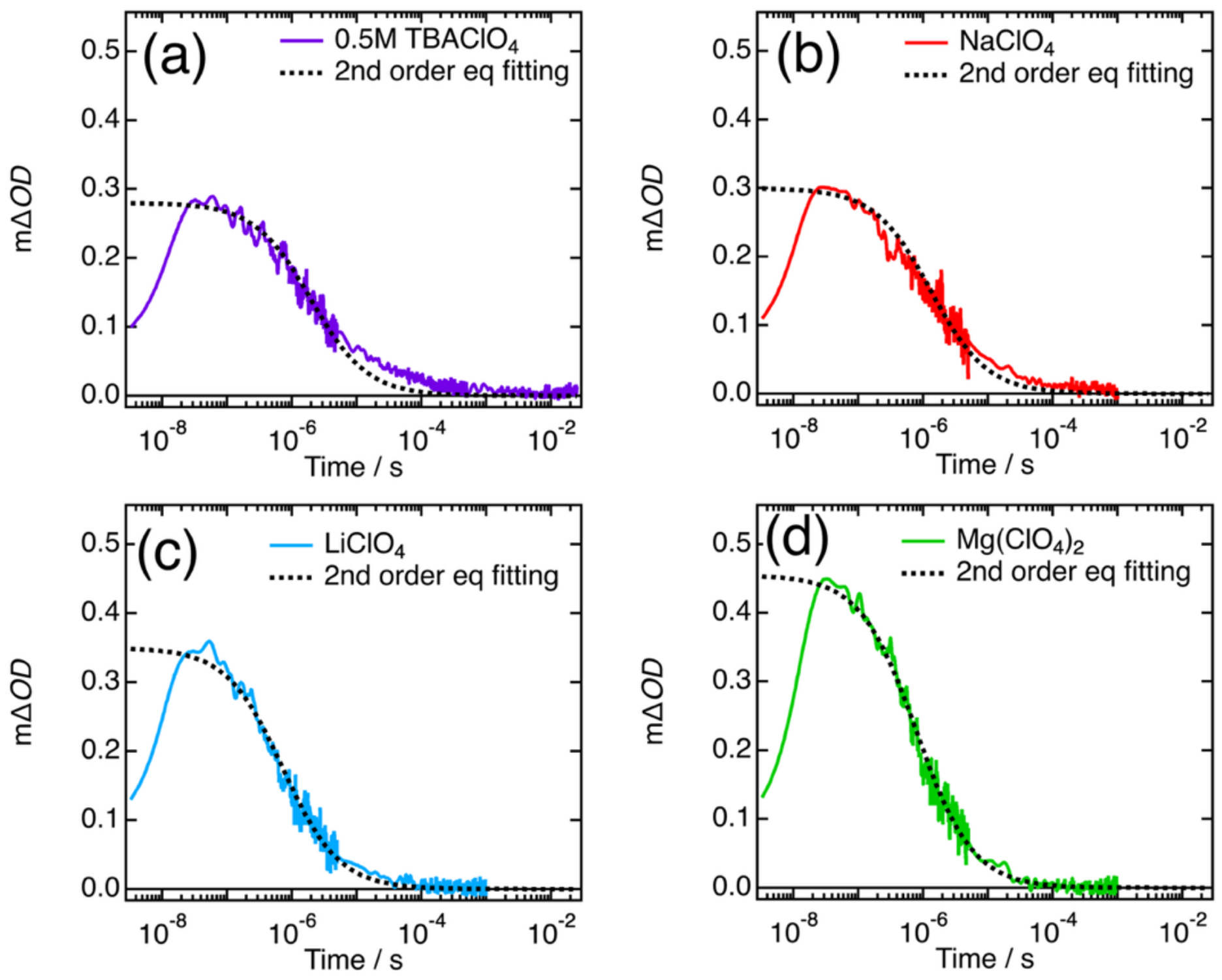

Figure S9. TA decays of MK-2 sensitized TiO_2 electrodes immersed in 0.5 M $TBAClO_4$ (a), $NaClO_4$ (b), $LiClO_4$ (c), and $Mg(ClO_4)_2$ (d) in acetonitrile. The excitation light intensity was the same for these measurements.

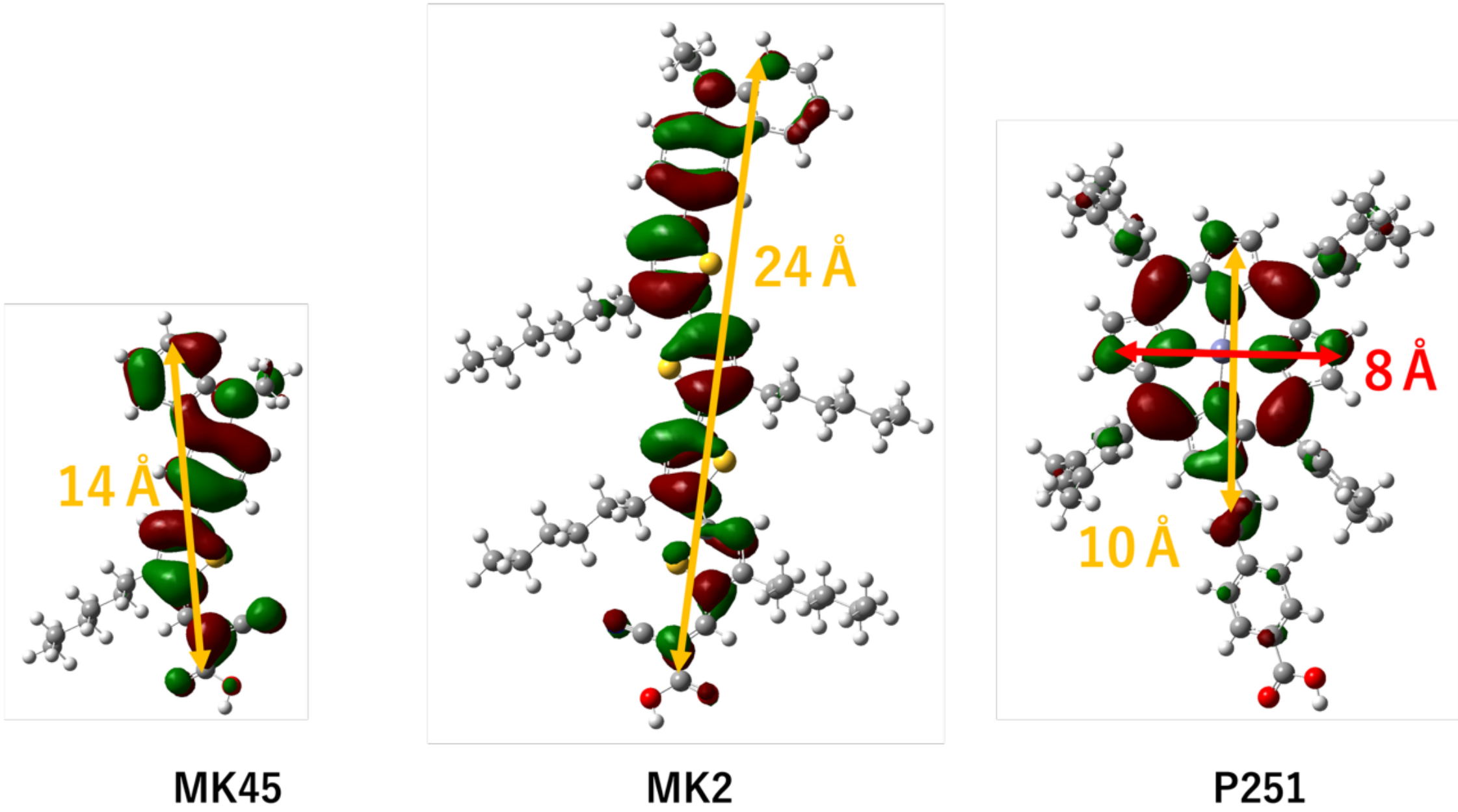

Figure S10. Highest occupied molecular orbitals of MK-45, MK-2, and P251 dyes. Structures were optimized by DFT using B3LYP/6-31g(d).

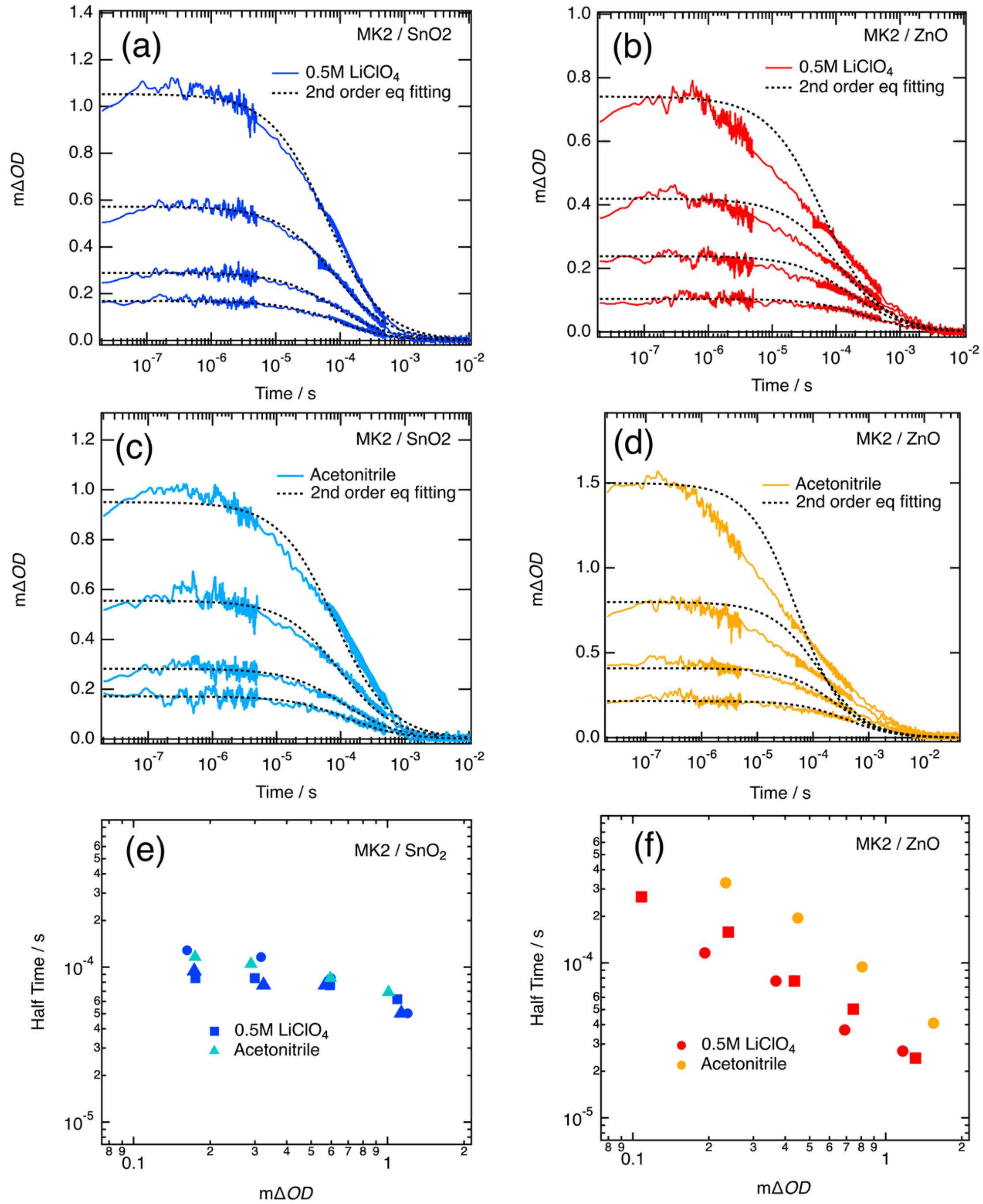

Figure S11. TA decays of MK-2 sensitized (a) SnO_2 and (b) ZnO electrodes immersed in 0.5 M $LiClO_4$ in acetonitrile, and (c) SnO_2 and (d) ZnO electrodes immersed in acetonitrile. Half-times of the TA decays as a function of initial ΔOD for SnO_2 (e) and ZnO (f). The different symbols with the same color indicate different samples prepared under identical conditions.

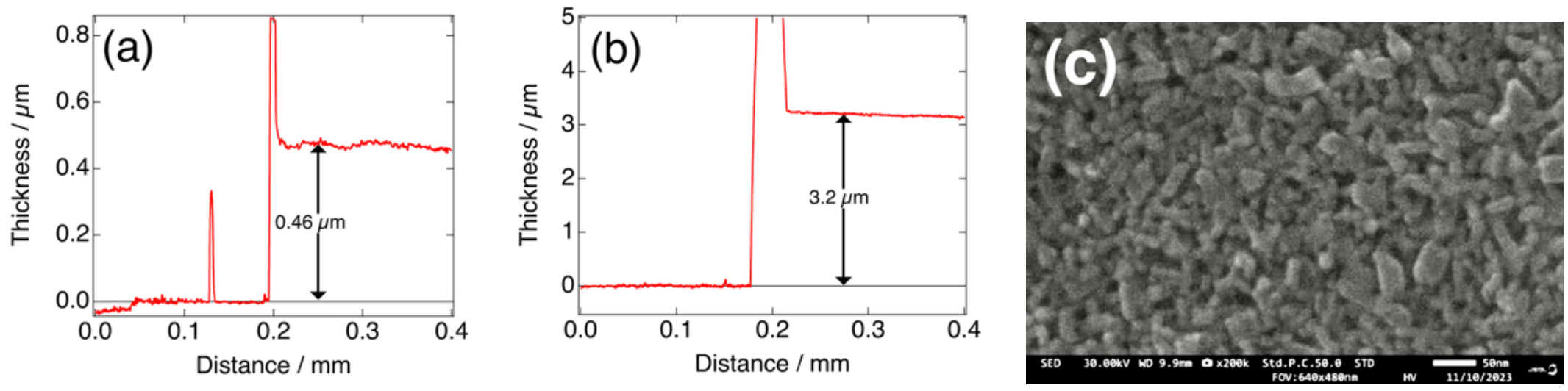

Figure S12. Surface profiles of the TiO_2 films used in the main experiments (a) and shown in Figure S1 (b), measured using an ET4000A surface profiler (Kosaka Laboratory Ltd., Japan; kindly provided by Prof. Kimura). Note that this is different from the surface profiler used to measure the thickness for this manuscript. (c) FE-SEM image of the mesoporous TiO_2 film (PST-18NR).

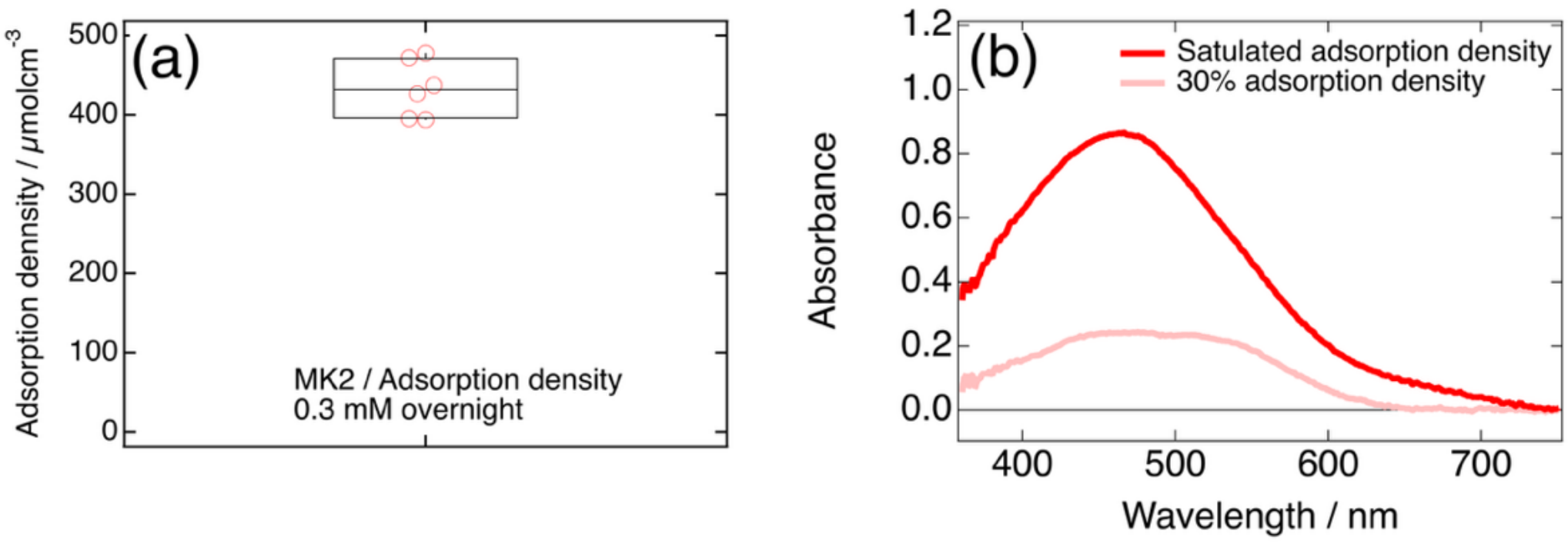

Figure S13. Saturated adsorption densities of MK-2 on several TiO_2 electrodes prepared under identical conditions. The median value is 430 µmol cm^{-3}. (b) Absorption spectra of the saturated and low adsorption density samples used in Fig. 4(d).

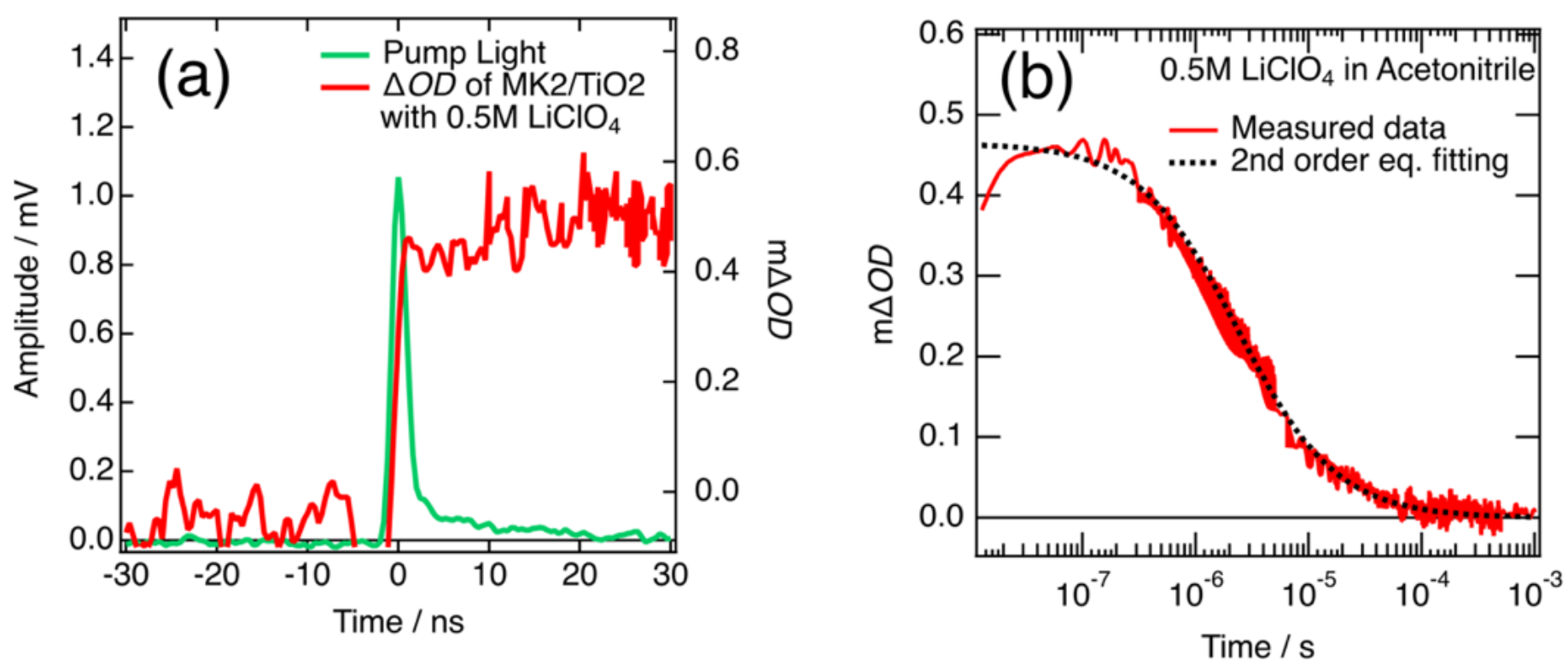

Figure S14. (a) The profile of the pump laser pulse and the rise of the transient absorption signal. (b) TA decay of MK-2 sensitized TiO_2 electrodes fabricated on glass, immersed in 0.5 M $LiClO_4$ in acetonitrile when the excitation laser intensity was relatively low.
